# Intermittent hypoxia training enhances Aβ endocytosis by plaque associated microglia via VPS35-dependent TREM2 recycling in murine Alzheimer’s disease

**DOI:** 10.1186/s13195-024-01489-6

**Published:** 2024-06-03

**Authors:** Xueting Wang, Yuqi Xie, Xiaoyang Fan, Xiaomei Wu, Dan Wang, Li Zhu

**Affiliations:** Institute of Special Environmental Medicine, Co-Innovation Center of Neuroregeneration, Nantong University, No.9, Seyuan Road, Chongchuan District, Nantong, Jiangsu 226009 China

**Keywords:** Alzheimer’s disease, Plaque-associated microglia, Beta-amyloid endocytosis, VPS35, TREM2 recycling, TFEB

## Abstract

**Background:**

Beta-amyloid (Aβ) deposition in the brain parenchyma is a crucial initiating step in the amyloid cascade hypothesis of Alzheimer’s disease (AD) pathology. Furthermore, dysfunction of plaque-associated microglia, also known as disease-associated microglia (DAM) has been reported to accelerate Aβ deposition and cognitive impairment. Our previous research demonstrated that intermittent hypoxia training (IHT) improved AD pathology by upregulating autophagy in DAM, thereby enhancing oligomeric Aβ (oAβ) clearance. Considering that oAβ internalization is the initial stage of oAβ clearance, this study focused on the IHT mechanism involved in upregulating Aβ uptake by DAM.

**Methods:**

IHT was administered to 8-month-old APP/PS1 mice or 6-month-old microglial vacuolar protein sorting 35 (VPS35) knockout mice in APP/PS1 background (MG VPS35 KO: APP/PS1) for 28 days. After the IHT, the spatial learning-memory capacity of the mice was assessed. Additionally, AD pathology was determined by estimating the nerve fiber and synapse density, Aβ plaque deposition, and Aβ load in the brain. A model of Aβ-exposed microglia was constructed and treated with IHT to explore the related mechanism. Finally, triggering receptor expressed on myeloid cells 2 (TREM2) intracellular recycling and Aβ internalization were measured using a fluorescence tracing technique.

**Results:**

Our results showed that IHT ameliorated cognitive function and Aβ pathology. In particular, IHT enhanced Aβ endocytosis by augmenting the intracellular transport function of microglial TREM2, thereby contributing to Aβ clearance. Furthermore, IHT specifically upregulated VPS35 in DAM, the primary cause for the enhanced intracellular recycling of TREM2. IHT lost ameliorative effect on Aβ pathology in MG VPS35 KO: APP/PS1 mice brain. Lastly, the IHT mechanism of VPS35 upregulation in DAM was mediated by the transcriptional regulation of VPS35 by transcription factor EB (TFEB).

**Conclusion:**

IHT enhances Aβ endocytosis in DAM by upregulating VPS35-dependent TREM2 recycling, thereby facilitating oAβ clearance and mitigation of Aβ pathology. Moreover, the transcriptional regulation of VPS35 by TFEB demonstrates a close link between endocytosis and autophagy in microglia. Our study further elucidates the IHT mechanism in improving AD pathology and provides evidence supporting the potential application of IHT as a complementary therapy for AD.

**Graphical abstract:**

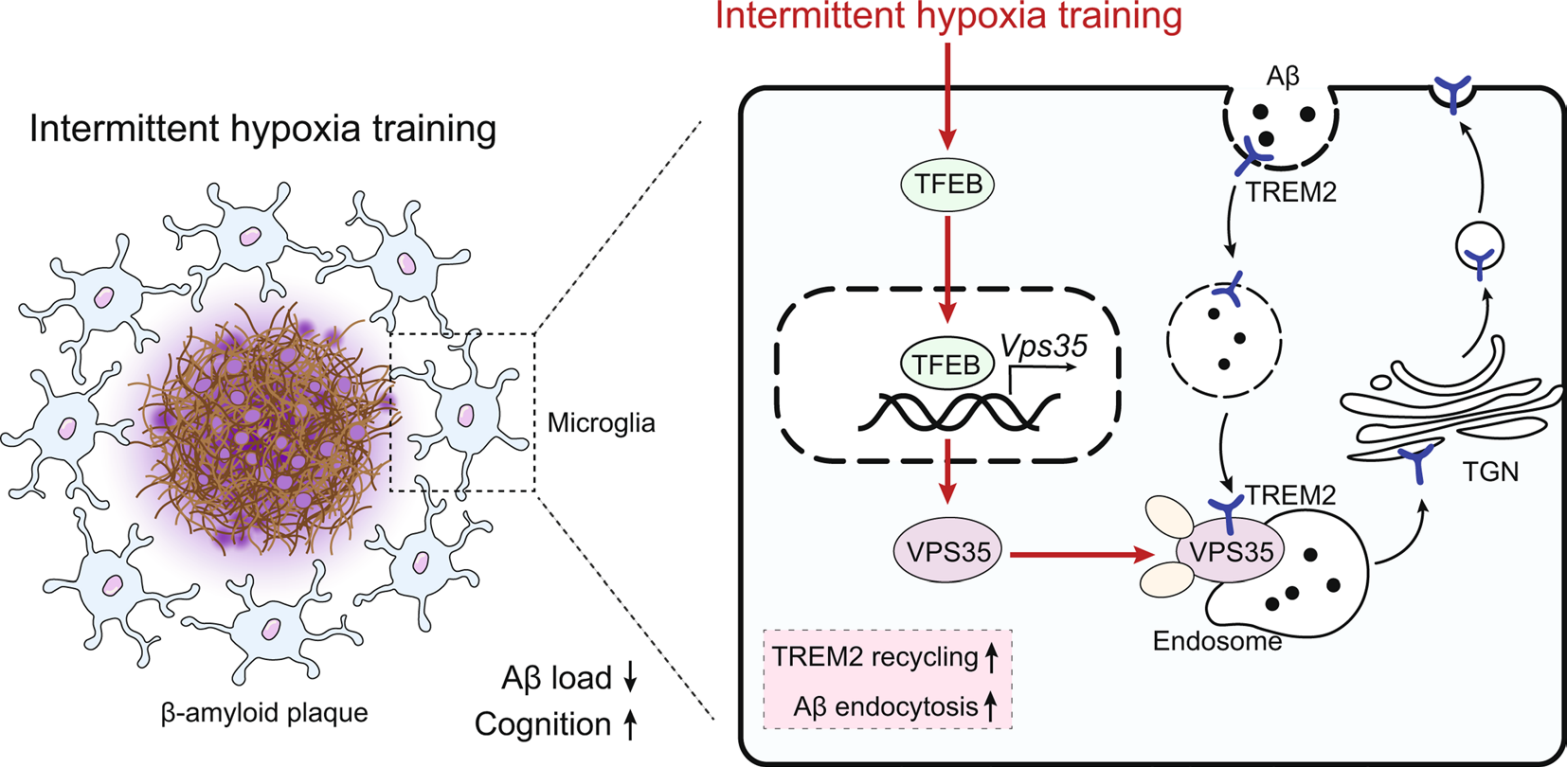

**Supplementary Information:**

The online version contains supplementary material available at 10.1186/s13195-024-01489-6.

## Introduction

Alzheimer’s disease (AD) is a neurodegenerative disorder exhibiting a high incidence in older adults, with a global incidence of 55 million people as of 2021. Beta-amyloid (Aβ) plaque deposition and neurofibrillary tangles in the brains of patients with AD trigger neuronal apoptosis, thereby causing dementia [1]. Therefore, research on AD pathogenesis has been focused on the Aβ cascade deposition theory and tau protein abnormality [2]. Recently, several monoclonal antibody drugs directed against Aβ have shown positive outcomes in AD treatment [3–5], indicating that reducing the toxic Aβ load in the brain is beneficial in AD treatment, particularly to improve cognitive function. The Aβ cascade hypothesis suggests that rapid AD development is associated with imbalanced Aβ production-clearance, i.e., an imbalance in Aβ production and clearance, ultimately triggering an overload of brain Aβ [6]. The amyloid precursor protein (APP) synthesized by neurons is sheared by β-/γ-secretase to generate Aβ monomers. The Aβ monomers then rapidly aggregate into Aβ oligomers to form Aβ fibers [7]. The adequate clearance of Aβ from the brain is essential for maintaining Aβ homeostasis [8]. Lonidamine ameliorates AD pathology by increasing ATP in microglia in the brain of AD mice and enhancing Aβ uptake by microglia [9]. A selective p38α/β MAPK inhibitor effectively enhances Aβ clearance by microglia and reduces Aβ deposition in the brain of 5XFAD mice [10]. Microglial phagocytosis is a vital mode of Aβ clearance [11, 12]. Microglia are activated and recruited by Aβ plaques to form plaque-associated microglia, which was wildly known as disease-associated microglia (DAM) [13]. DAM have been found to upregulate triggering receptor expressed on myeloid cells 2 (TREM2), Clec7a, and ApoE [14, 15] as well as engulf and autophagically degrade Aβ via LC3-associated endocytosis (LANDO) in the early stages of AD [16]. However, excessive Aβ loading in late-stage AD impairs LANDO in DAM, leading to reduced Aβ uptake and clearance by DAM that further exacerbates Aβ overload in the brain [16]. Based on this mechanism, lonidamine, a hexokinase inhibitor, has been shown to enhance Aβ uptake by DAM, attenuate Aβ load, and improve AD pathology [9]. Therefore, targeting DAM for Aβ clearance has good potential in AD treatment.

Microglial TREM2-mediated endocytosis of Aβ is the initial and critical step in Aβ clearance by microglia [17–19]. TREM2 is upregulated in the brains of patients with AD and AD model mice [20], with this compensatory upregulation being possibly related to the impaired microglial TREM2 function in this disease condition. The TREM2^R47H^ variant exhibits a marked decrease in TREM2 membrane expression and increased lysosomal localization, leading to a loss of responsiveness to Aβ in microglia and induction of late-onset AD [21–23]. Previous studies have shown that the abnormal intracellular localization of TREM2^R47H^ may contribute to Aβ overloading [24, 25], implying that alterations in the TREM2 localization rather than its intracellular concentration have a greater effect on its function.

Vacuolar protein sorting 35 (VPS35) is a pivotal component of the Retromer complex. The Retromer, first identified in yeast, is a heterotrimeric pentameric protein complex that comprises highly sequence-conserved core structures of VPS35, VPS26, and VPS29, along with sorting nexins (SNXs) [26]. Following the entry of the Sortilin-related receptor (SORLA) into the cell via the endosomal pathway, SNXs (e.g., SNX27) selectively bind to SORLA via the N-terminal end. Subsequently, the VPS35-centered Retromer facilitates the translocation of SORLA from endosomes to the cell surface [27]. Studies have demonstrated that Retromer dysfunction is associated with a variety of neurodegenerative diseases. For instance, VPS35 mutations induced by aspartic acid asparagine (D620N) have been linked to familial and idiopathic Parkinson’s disease [28]. VPS35 deficiency leads to Retromer dysfunction, resulting in the incorrect sorting of SORLA into lysosomes for degradation [29]. Similarly, SNX27 deficiency affects the sorting of the Aβ receptor, SORLA, back to the membrane, thereby exacerbating Aβ pathology [30]. VPS35 deficiency reduces the Aβ uptake by microglia and aggravates cognitive dysfunction in AD mice [31, 32]. Moreover, TREM2 recycling depends on VPS35, with VPS35 deficiency or TREM2 mutation leading to decreased TREM2 membrane expression and increased lysosomal localization [24, 25]. Additionally, brain VPS35 was found to be significantly reduced in patients with AD and AD model mice [33, 34], highlighting the essential role of VPS35 in DAM. All these findings emphasize that targeting VPS35 can be an effective treatment approach for AD.

Intermittent hypoxia training (IHT) is an oxygen conditioning technique that improves the body’s aerobic metabolism and facilitates its adaptation to the hypoxic environment by inducing moderate hypoxia in the body [35, 36]. IHT has been found to effectively upregulate autophagy, which in turn ameliorates cardiac systolic dysfunction and myocardial injury in rats [37] as well as significantly alleviates cognitive impairment and AD in mice [35]. Our previous study demonstrated that IHT improved the Aβ degradation activity of DAM by upregulating the autophagic lysosomal process and reducing the Aβ load in the brain [38]. However, Aβ degradation by DAM initially requires the effective uptake of Aβ [39] and whether IHT improves Aβ uptake via DAM remains unclear. We have earlier identified that IHT activates the nuclear translocation of transcription factor EB (TFEB) in DAM, along with activating downstream autophagy-related genes transcription [38]. TFEB, as a key transcription factor regulating autophagy lysosomal function, is essential for the production and clearance of Aβ in the brain. Fumiko Yamamoto et al. demonstrated that upregulation of TFEB in neurons effectively reduces Aβ production and significantly decreases Aβ1–42 by enhancing the autophagy-lysosomal process [40]. Starvation-induced TFEB activation has been reported to enhance autophagy in HeLa cells via the transcriptional regulation of VPS35 ^41^. However, whether IHT affects the function of DAM by modulating TREM2 remains unknown. Here, we hypothesized that IHT upregulated VPS35 by activating the nuclear TFEB in DAM, which resulted in a further upregulation of TREM2-mediated endocytosis of Aβ by DAM and subsequent reduction in Aβ load in the brain. This study aimed to elucidate the relationship between TFEB and microglial endocytosis and to verify the close link between endocytosis and autophagy. Further, we attempted to explore the molecular mechanisms involved in IHT-associated amelioration of AD pathology and to provide supporting data for advancing the clinical application of IHT in AD.

## Materials and methods

### Animals

APP/PS1 mice (B6C3-Tg[APPswe, PSEN1de9]85Dbo/J [005864]) and CX3CR1^Cre − ER^ mice (021160, B6.129P2[Cg]-Cx3cr1tm2.1[cre/ERT2]Litt/WganJ) were purchased from Nanjing Junke Bioengineering Corporation, Ltd. (Nanjing, China; certification number: SCXK 2020-0009). VPS35^fl/fl^ mice (C57BL/6 N-Vps35 tm1c[EUCOMM]Hmgu/Cmsu) constructed by CAM-SU GRC were provided by Professor Tong Liu from Nantong University. The VPS35^fl/fl^ mice were crossed with the CX3CR1^Cre − ER^ mice as previously reported [42], followed by crossing with the APP/PS1 mice to generate VPS35^fl/fl^: CX3CR1^Cre − ER^: APP/PS1 mice. At postnatal days 45, 95, and 150, the VPS35^fl/fl^: CX3CR1^Cre − ER^: APP/PS1 mice and their littermate control VPS35^fl/fl^: APP/PS1 mice were intraperitoneally injected with 100 mg/kg of tamoxifen (Sigma, #T5648) for 5 days. Mice showing a successful knockout of microglial VPS35 were designated as MG VPS35 KO: APP/PS1 mice and their littermate controls were denoted as VPS35 ^fl/fl^:TG mice. Experimental animals were maintained at 23 ± 2 °C and 45–60% humidity with a standard 12/12-h light/dark cycle. All study protocols involving animal experiments were reviewed and approved by the Animal Care and Use Committee of Nantong University and the Jiangsu Province Animal Care Ethics Committee (approval ID: SYXK[SU]2007-0021).

### Animal experimental design

*IHT treatment*. IHT treatment was performed as described previously [38]. Briefly, 8-month-old APP/PS1 (TG) mice or 6-month-old MG VPS35 KO: APP/PS1 were placed in a 60 cm × 30 cm × 25 cm chamber. The oxygen concentration in the chamber was initially reduced to 8% within 30 s and maintained for 8 min by introducing compressed nitrogen. Compressed oxygen was then introduced to restore the oxygen concentration to 20% within 30 s and sustained for 8 min. A total of 10 cycles of this hypoxia-normoxia exposure was conducted daily for 28 days between 09:00 a.m. and 11:00 a.m.

*TFEB activator 1 (TA1) administration*. TA1 (MedChemExpress, HY-135,825, CAS: 39777-61-2, 99.69% purity) was dissolved in DMSO to a concentration of 25 mg/ml and further diluted 10-fold with corn oil to 2.5 mg/ml for animal treatment. TA1 at 10 mg/kg/day or vehicle (corn oil) was orally administered to the 6-month-old APP/PS1 mice for 3 months [43].

*Eltrombopag (EO) administration*. EO (MedChemExpress, HY-15,306, CAS: 3 496775-61-2, 99.73% purity) was dissolved in DMSO to a concentration of 30 mg/ml, followed by 10-fold dilution with corn oil to 3 mg/ml for animal treatment. EO (30 mg/kg/day) or vehicle (corn oil) was injected intraperitoneally in the 8-month-old mice for 14 days [44].

After these treatments, the mice were euthanized and perfused with 0.9% saline via the left ventricle to remove the blood.

### Morris water maze (MWM) test

MWM test for assessing spatial learning-memory behavior was conducted as previously described by Zha et al. [45]. In this test, a 150-cm circular pool was divided into four equal quadrants, namely the northeast, southeast, southwest, and northwest quadrants. Visual cues were placed on the walls around the maze to facilitate the spatial learning of the platform’s location. The water used in the maze was made opaque with a non-toxic, white pigment and maintained at a temperature of 21 ± 1 °C. During the training period (4 times/day for 5 days), a 10-cm diameter circular platform was placed in the middle of the southwest quadrant at 1.5 cm below the water surface. Individual mice were then allowed to swim freely for 60 s to find the platform and stay on it for 20 s. On the probe test day (the 6th day), the platform was removed. The mice were released in the northeast quadrant and allowed to swim freely for 60 s. Video recordings were utilized to analyze and record the swimming trajectory, along with measurement of escape latency (i.e., time to find the escape platform) of the mice and crossing frequency to the target quadrant.

### Primary microglia culture and construction of Aβ-exposed microglia

*Primary microglia culture.* Based on earlier established protocols [46–48], primary microglia were obtained from the cerebral cortices of 2-day-old C57BL/6 mice. In this procedure, single-cell suspensions of brain tissue were prepared by digestion in 0.05% trypsin, followed by culturing in DMEM-F12 medium (Thermo Fisher, 11,320,033) containing 10% fetal bovine serum (Celligent, CG0430B), GlutaMAX supplement (Thermo Fisher, 35,050,079), 5 ng/ml of granulocyte-macrophage colony-stimulating factor (STEMCELL Technologies, 78,017), and penicillin/streptomycin (100 U/ml and 100 mg/ml, respectively) at 37 °C in a 5% CO_2_ humidified incubator. After 10 days of growth, the mixed cell population was dominated by astrocytes, forming a fused trophoblast. Microglial cells gradually proliferated and floated in the supernatant, and they were harvested on the 14th day.

*Construction of Aβ-exposed microglia.* Lyophilized Aβ1–42 (AnaSpec, AS-20,276) was dissolved in PBS and incubated overnight at 4 °C to form oligomers (oAβ) [49]. Finally, primary microglia or BV2 cells were treated with 1 µM of oAβ for 12 h.

### Cell transfection and treatment

*Lentivirus transfection in primary microglia.* Microglial VPS35 was knocked out by transfecting lentivirus expressing Cre-GFP into trophoblast cells obtained from Vps35^fl/fl^ mice. Additionally, TFEB was silenced by transfecting lentivirus expressing *shTfeb* (target sequence: GCGGCAGAAGAAAGACAATCA) into trophoblast cells isolated from C57BL/6 mice. After 7 days of primary culture, the cells were incubated with 8 × 10^7^ TU of lentiviruses per 25 cm^2^ culture flask and allowed to grow until microglia production. The lentivirus transfection efficiency was approximately 50% in the harvested microglia [38].

*Construction of shTfeb BV2 cells*. BV2 cells were transfected with *shTfeb*-expressing lentivirus at multiplicity of infection = 10. The GFP^+^ cells were then isolated via flow cytometry and cultured to form single-cell clones.

*IHT treatment.* Cultured cells were placed in the same intermittent hypoxia chamber used for the experimental mice. The cells were exposed to 21% oxygen and 8% oxygen (30 s for the oxygen to jump between the two concentrations) for 8 min cycles. The viability of cells after IHT treatment was measured by a Cell Counting Kit-8 (CCK-8, HY-K0301, MedChemExpress).

*TA1 treatment*. TA1 was diluted to 1 mM in DMSO and directly added to the culture medium to yield a working concentration of 1 µM.

### Oligomeric Aβ-555 endocytosis and TREM2 or TFR1 recycling assays

*Aβ-555 endocytosis assay.* To test the effect of IHT or TA1 treatment on Aβ-exposed microglia internalized oAβ, cells were incubated with 1 µg/ml oligomeric Aβ1–42, HiLyte™ Fluor 555 (Aβ-555) (AnaSpec, AS-60480-01) for 30 min and fixed with 4% paraformaldehyde, followed by counterstaining with DAPI. Since non-fluorescently labeled oAβ was used in the construction of the Aβ-exposed microglia, the red fluorescence observed under the microscope were all internalized oAβ in the cells within 30 min.

*TREM2 or TFR1 internalization and recycling assays.* After IHT treatment, primary microglia were incubated in DMEM-F12 medium containing sheep anti-TREM2 antibodies (R&D Systems, AF1729) or mouse anti-TFR1 antibodies (Thermo Fisher, 13-6800) for 1 h at 4 °C. The microglia were then washed with a pre-cooled DMEM-F12 medium to remove unbound antibodies. Subsequently, the cells were incubated with DMEM-F12 medium containing 10% fetal bovine serum for 30 min at 21% O_2_, 5% CO_2_, 37 °C to stimulate TREM2 or TFR1 internalization. Cells were then washed with wash solution (hydrochloric acid added to DMEM/F12 medium to reduce the pH to 2.0) for 5 s to remove surface antibodies, and then the wash solution was immediately removed with fresh DMEM/F12 medium for 2 min to restore the extracellular pH environment. In the internalization assay, the cells were directly fixed and labeled with anti-sheep Cy3 antibodies (Jackson ImmunoResearch, 713-165-174) or anti-mouse Alexa 488 antibodies (Jackson ImmunoResearch, 715-545-150) (Fig. 2A). In the case of the recycling assay, the cells were further incubated with DMEM-F12 medium containing 10% fetal bovine serum for 60 min at 21% O_2_, 5% CO_2_, 37 °C. The live cells were then probed by incubating in DMEM-F12 medium containing anti-sheep Cy3 antibodies for 1 h at 4 °C, followed by fixation (Fig. 2D).

### Immunofluorescence staining

Brain tissue sections or cultured cells were fixed and subsequently permeabilized with 0.5% Triton X-100. Next, the samples were blocked in 10% donkey serum, followed by incubation with the primary antibodies at 4 °C overnight. Secondary antibodies were then employed to visualize the binding of the primary antibodies. Lastly, the samples were counterstained with DAPI, and confocal microscopy (Leica SP8 confocal microscope) was performed to capture the fluorescence images. The antibodies utilized for immunofluorescence staining are listed in Table 1. Individual Iba1-positive cells were selected and the average fluorescence intensity of VPS35, TREM2 and TFEB in the selected cells was counted using FIJI software. The data were subsequently normalized with Nor-TG.

**Table 1:** Antibody information

Name	Catalog number	Company
Anti-Aβ,1–16 Antibody	SIG-39,300	Biolegend
Anti-VPS35	sc-374,372	Santa cruz
Anti-TFEB	13372-1-AP	Proteintech
Anti-Iba1	ab5076	Abcam
Anti-TREM2	AF1729	R&D system
Anti-LAMP1	sc-20,011	Santa cruz
Anti-Rab5	Sc-46,692	Santa cruz
Anti-PSD95	3409T	Cell Signaling Technology
Anti-NeuN	MAB377	Millipore
Anti-β-actin	66009-1-lg	Proteintech
Donkey anti-Sheep Alexa 647	ab150179	Abcam
Donkey anti-Sheep Alexa 488	ab150177	Abcam
Donkey Anti-Sheep Cy3	713-165-147	Jackson Immuno Research
Donkey anti-Goat Cy3	705-165-003	Jackson Immuno Research
Donkey anti-Mouse Alexa 488	715-545-150	Jackson Immuno Research
Donkey anti-Mouse Alexa 555	ab150110	Abcam
Donkey anti-Rabbit Alexa 488	ab150073	Abcam
Donkey anti-Rabbit Alexa 555	ab150074	Abcam

### RNA isolation and qRT-PCR

Purified total RNA from the brain tissue or cells underwent reverse transcription using the HiScript III 1st Strand cDNA Synthesis Kit (Vazyme Biotech, R312-02). qRT-PCR was performed for a total of 40 cycles at 95 °C for 10 s, 60 °C for 30 s, and 72 °C for 20 s, according to the instructions in the AceQ qPCR SYBR Green Master Mix (Vazyme Biotech, Q141-02). The relative amount of gene expression was calculated using ΔCt values. The primers used for qRT-PCR are provided in Table 2.

**Table 2:** Primer information

Name	Direction	Sequence (5′—3′)
*Trem2*	Forward	TGGAACCGTCACCATCACTC
	Reverse	GAGGTGACCCACAGGATGAA
*Vps35*	Forward	CGGACCTCTATGCTGTCACC
	Reverse	CATCCGCACCCAGAGCTTAT
*Tfeb*	Forward	CGCCTGGAGATGACTAACAAGC
	Reverse	GGCAACTCTTGCTTCACCACCT
*Vps35 (-155/118)*	Forward	GCCGCAGCGTTGTACTATTC
	Reverse	AGGAAAACGTGGACTGCGA
*Vps35 (148/278)*	Forward	AGGAGGCCCTGCATTTCGAT
	Reverse	GGGTAACGACCAAATTCTGCG
*Vps35(341/660)*	Forward	TGCGTCAGACTCTTGTACGG
	Reverse	GAAAGCAGGAGTCCTTCGGG
*Vps26*	Forward	AAGAGGCTAGAGCATCAAGGA
	Reverse	TTCTCCAGGCAAGGCTAGTTC
*Vps29*	Forward	CTGCACCAAGGAGAGCTACG
	Reverse	TCAGACCGATCTTGAACTGGC
*Snx27*	Forward	GGTGTCAGACATTGAGCATGGC
	Reverse	AGCCATAGTGCCGCAGAGTTTG
*Actb*	Forward	CATCCGTAAAGACCTCTATGCCAAC
	Reverse	ATGGAGCCACCGATCCACA

### Protein isolation and Western blotting

The samples were lysed in RIPA buffer, and their protein concentrations were determined via the bicinchoninic acid assay. The proteins in the remaining lysate were isolated and transferred to polyvinylidene fluoride membranes for primary antibody hybridization. Finally, HRP-conjugated secondary antibodies were utilized to visualize the binding of the primary antibodies.

### Statistical analysis

The fluorescent signal intensity on microscopy imaging and the grayscale values of the Western blotting protein bands were calculated using FIJI software (National Institutes of Health). Data analyses were performed using GraphPad Prism version 8 software. Statistical assessment was conducted using Student’s *t*-test and two-way ANOVA, followed by Dunnett’s multiple comparisons test. Data were presented as mean ± SEM. The significance levels for all graphs were as follows: *p < 0.05, **p < 0.01, and ***p < 0.001. n.s. indicated no statistical difference.

## Results

### IHT enhances TREM2 recycling in DAM and ameliorates Aβ pathology

**Fig. 1:**
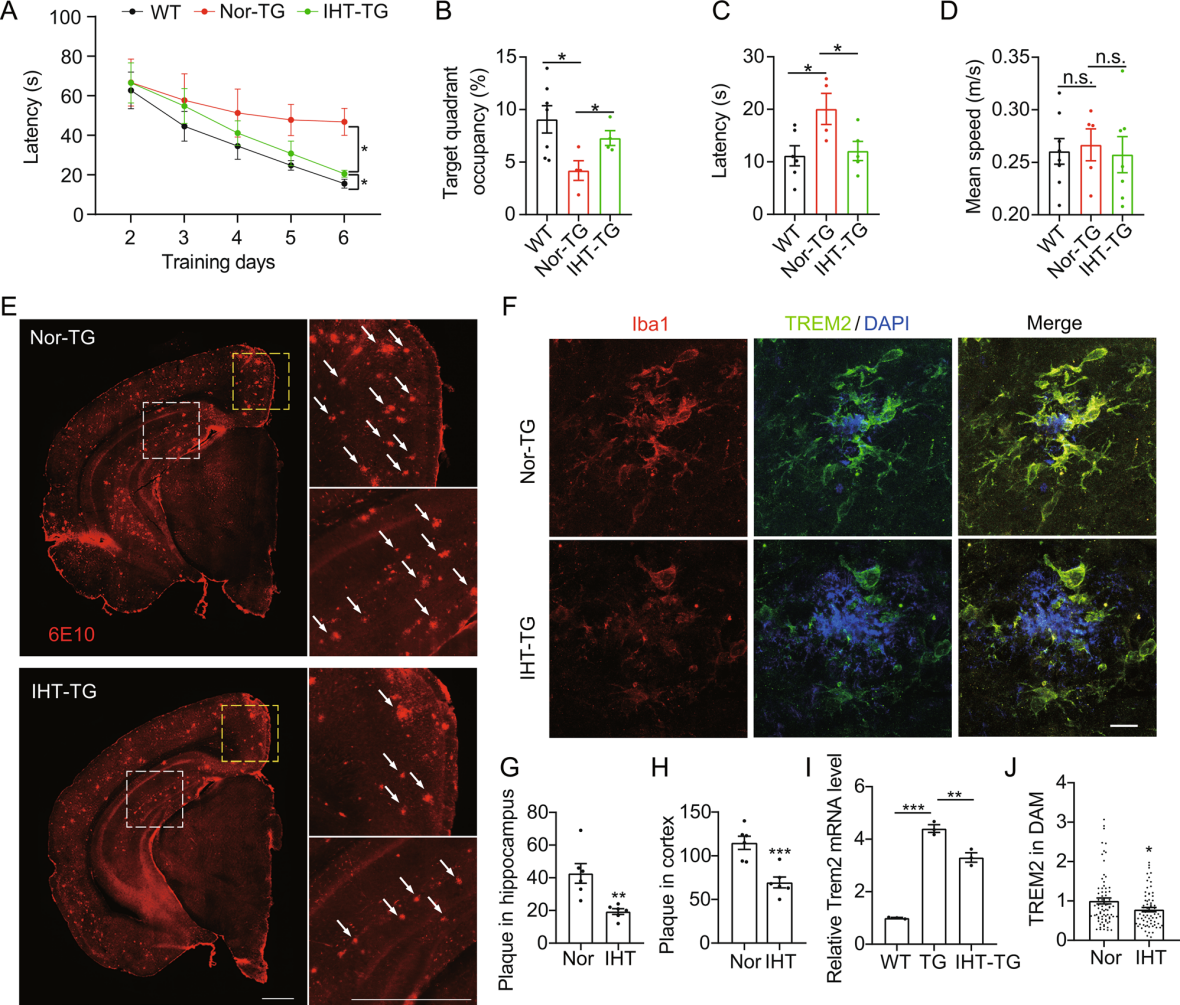
IHT downregulates TREM2 in DAM and reduces Aβ load in brains of 9-month-old APP/PS1 mice. (**A**) Escape latency of the mice to reach the target platform during the MWM training period. (**B**) The ratio of dwelling time in the target quadrant compared to all quadrants during the MWM probe test. (**C**) The escape latency of mice to reach the target quadrant during the MWM probe period. (**D**) The average swimming speed of mice during the MWM probe test (*n* = 7). (**E**) Brain sections were probed with 6E10 antibodies, and microscopy images of the Aβ plaques were obtained. White arrows indicate plaques. Scale bar = 1 mm. (**F**) Brain sections were incubated with anti-TREM2 and anti-Iba1 antibodies, and microscopy images of DAM in the CA1 region were acquired. Scale bar = 10 μm. (**G**) Number of Aβ plaques in the hippocampus in panel **E** (*n* = 6). (**H**) Number of Aβ plaques in the cortex in panel **E** (*n* = 6). (**I**) *Trem2* expression in the CA1 region was measured using qRT-PCR (*n* = 3). (**J**) TREM2 intensity in the DAM in panel **F** (six mice in each group, with 15 cells per mouse). *p < 0.05, **p < 0.01, and ***p < 0.001 by Student’s *t*-test. Nor, normoxia; IHT, intermittent hypoxia training; WT, wild type; TG, APP/PS1

After 28 days of IHT, spatial learning and memory were significantly improved in 9-month-old APP/PS1 mice (Fig. 1A to D). Correspondingly, the number of brain Aβ plaques was significantly reduced (Fig. 1E, G and H), consistent with our previous findings [38]. Additionally, we have earlier demonstrated that IHT attenuates Aβ levels by inducing TFEB-mediated autophagy in microglia [38]. Considering that Aβ uptake is the initial step in Aβ clearance by microglia, we investigated whether IHT affected Aβ endocytosis in the microglia. Given that Aβ endocytosis depends on microglial TREM2 [50], we further examined TREM2 levels in the brains of mice that underwent IHT. As shown in Fig. 1I, TREM2 was significantly upregulated in the AD mice brains, in line with prior study results [51]. Conversely, TREM2 was significantly downregulated at the mRNA level (Fig. 1I) as well as in DAM (Fig. 1F and J) after IHT treatment, suggesting that the IHT-associated promotion of Aβ uptake by microglia might not be achieved by increasing the biological level of TREM2

**Fig. 2:**
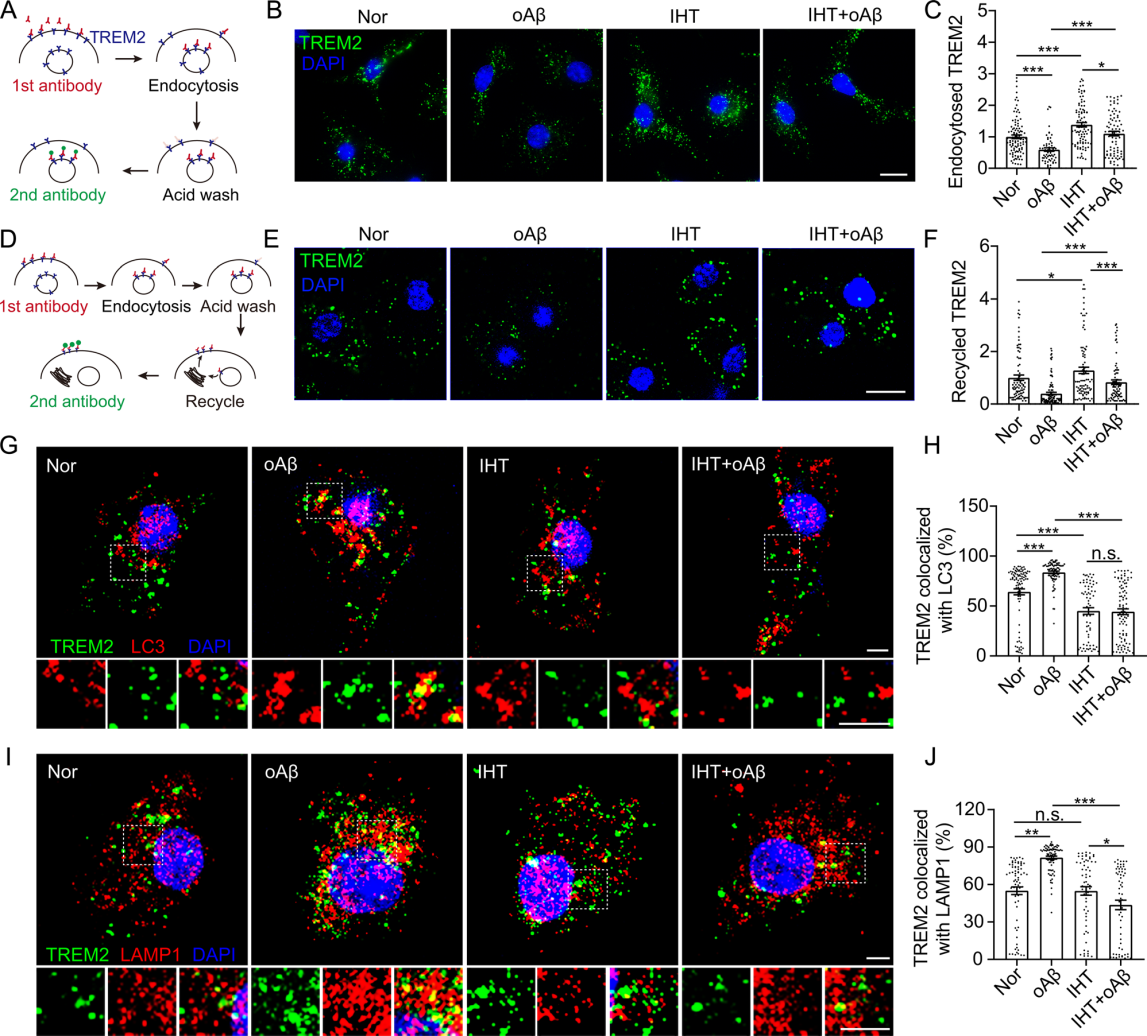
IHT enhances TREM2 recycling and Aβ endocytosis in Aβ-exposed microglia. (**A**) Flowchart of TREM2 internalization test. (**B**) After treatment with IHT, the TREM2 internalization assay was conducted in Aβ-exposed microglia, followed by the fixation of the cells and counterstaining with DAPI. Scale bar = 10 μm. (**C**) TREM2 intensity in Aβ-exposed microglia in panel **B** (*n* > 80). (**D**) Flowchart of TREM2 recycling test. (**E**) After treatment with IHT, the TREM2 recycling assay was performed in Aβ-exposed microglia. The cells were then fixed and counterstained with DAPI. Scale bar = 10 μm. (**F**) TREM2 intensity in Aβ-exposed microglia in panel **E** (*n* > 80). (**G**) After treatment with IHT, membrane TREM2 was labeled with anti-TREM2 antibodies in Aβ-exposed microglia and allowed to undergo internalization for 60 min. Subsequently, the cells were fixed and probed with anti-LC3 antibodies. Scale bar = 5 μm. (**H**) Colocalization ratio of TREM2 with LC3 in single cells in panel **H** via Manders’ colocalization coefficients (*n* > 80). (**I**) After treatment with IHT, membrane TREM2 was labeled with anti-TREM2 antibodies in Aβ-exposed microglia and allowed to undergo internalization for 60 min. Subsequently, the cells were fixed and probed with anti-LAMP1 antibodies. Scale bar = 5 μm. (**J**) Colocalization ratio of TREM2 with LAMP1 in single cells in panel **I** via Manders’ colocalization coefficients (*n* > 80. *p < 0.05, **p < 0.01, and ***p < 0.001 by two-way ANOVA. Nor, Normoxia; IHT, intermittent hypoxia training

Previous research has indicated that the effective intracellular recycling of TREM2, a specific receptor for Aβ, is required to maintain the Aβ uptake capacity of microglia [50]. Hence, we performed internalization and recycling experiments of TREM2 (Fig. 2A and D) to determine whether IHT positively affected the intracellular recycling of TREM2. We first verified that IHT had no significant effect on the viability of Aβ-exposed microglia (Supp. Figure 1). As depicted in Fig. 2B and C, TREM2 internalization was significantly reduced in Aβ-exposed microglia, and this TREM2 internalization was significantly upregulated after IHT. IHT also significantly improved TREM2 recycling in Aβ-exposed microglia (Fig. 2E and F). Consistently, IHT markedly attenuated autophagic degradation of TREM2 in in Aβ-exposed microglia (Fig. 2G to J). These results were in line with our previously report that IHT significantly reversed the impairment of oAβ endocytosis by Aβ-exposed microglia [38], suggesting that IHT enhanced Aβ endocytosis by augmenting the intracellular transport function of microglial TREM2, ultimately contributing to Aβ clearance.

### IHT specifically upregulates VPS35 expression in DAM

**Fig. 3:**
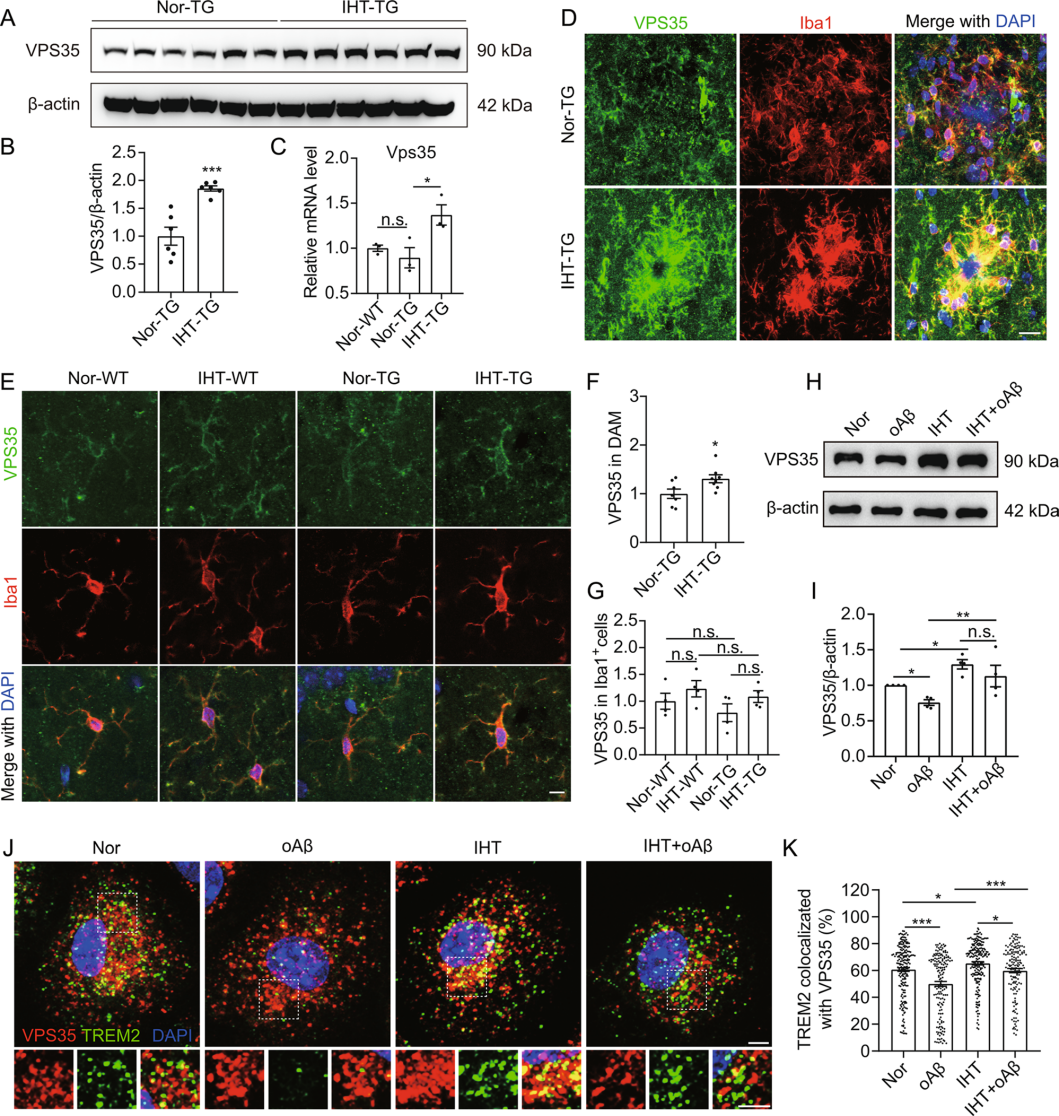
IHT upregulates VPS35 in DAM and Aβ-exposed microglia. (**A**) VPS35 expression in IHT-treated mice brain cortex was detected using Western blotting. **(B)** Grayscale values of the protein bands in panel **A** (*n* = 6). (**C**) *Vps35* expression in the CA1 regions was estimated via qRT-PCR (*n* = 3). (**D** and **E**) Brain sections were labeled with anti-VPS35 and anti-Iba1 antibodies, and microscopy images of DAM (**D**) and microglia not associated with plaques in CA1 region were captured (**E**). Scale bar = 10 μm. (**F**) The average fluorescence intensity of VPS35 in the Iba1^+^ cells of DAM in panel **D** (*n* = 7, each data point represents the average intensity in DAM of 6 plaques in each mouse brain). (**G**) VPS35 intensity in the Iba1^+^ cells in panel **E** (*n* = 4). (**H**) After treatment with IHT, VPS35 expression in Aβ-exposed microglia was detected using Western blotting. (**I**) Grayscale values of the protein bands in panel **H** (*n* = 4). (**J**) After treatment with IHT, membrane TREM2 was labeled with anti-TREM2 antibodies in Aβ-exposed microglia and allowed to undergo internalization for 30 min. Subsequently, the cells were fixed and probed with anti-VPS35 antibodies. Scale bar = 3 μm. (**K**) Colocalization ratio of TREM2 with VPS35 in single cells via Manders’ colocalization coefficients (*n* > 100). *p < 0.05, **p < 0.01, and ***p < 0.001 by Student’s *t*-test (**B**, **C** and **F**) or two-way ANOVA (**G**, **I**, and **K**). n.s. indicates no significant difference. Nor, normoxia; IHT, intermittent hypoxia training; WT, wild type; TG, APP/PS1

TREM2 recycling has been reported to depend on VPS35 ^24^. In our study, IHT was found to upregulate VPS35 in AD mice brains (Fig. 3A to C). In particular, IHT demonstrated a significant upregulation of VPS35 only in DAM (Fig. 3D and F) but not in microglia not associated with plaques (Fig. 3E and G). Correspondingly, in Aβ-exposed microglia, IHT promoted the expression of VPS35 but not VPS26, VPS29, or SNX27 (Fig. 3H and I, Supp. Figure 2), consistent with our in vivo results. Thus, the above findings indicated that IHT specifically upregulated VPS35 in amyloid-stimulated microglia. In addition, IHT significantly enhanced the intracellular recycling of TFR1 (transferrin receptor 1) (Supp. Figure 3), which has been reported as a cargo of VPS35-Retromer [52]. Since IHT upregulated the colocalization of VPS35 with TREM2 in Aβ-exposed microglia (Fig. 3J and K), implying that the IHT-induced improvement in TREM2 intracellular recycling in DAM might be related to VPS35 upregulation.

### IHT augments Aβ endocytosis by Aβ-exposed microglia via VPS35-dependent TREM2 recycling

**Fig. 4:**
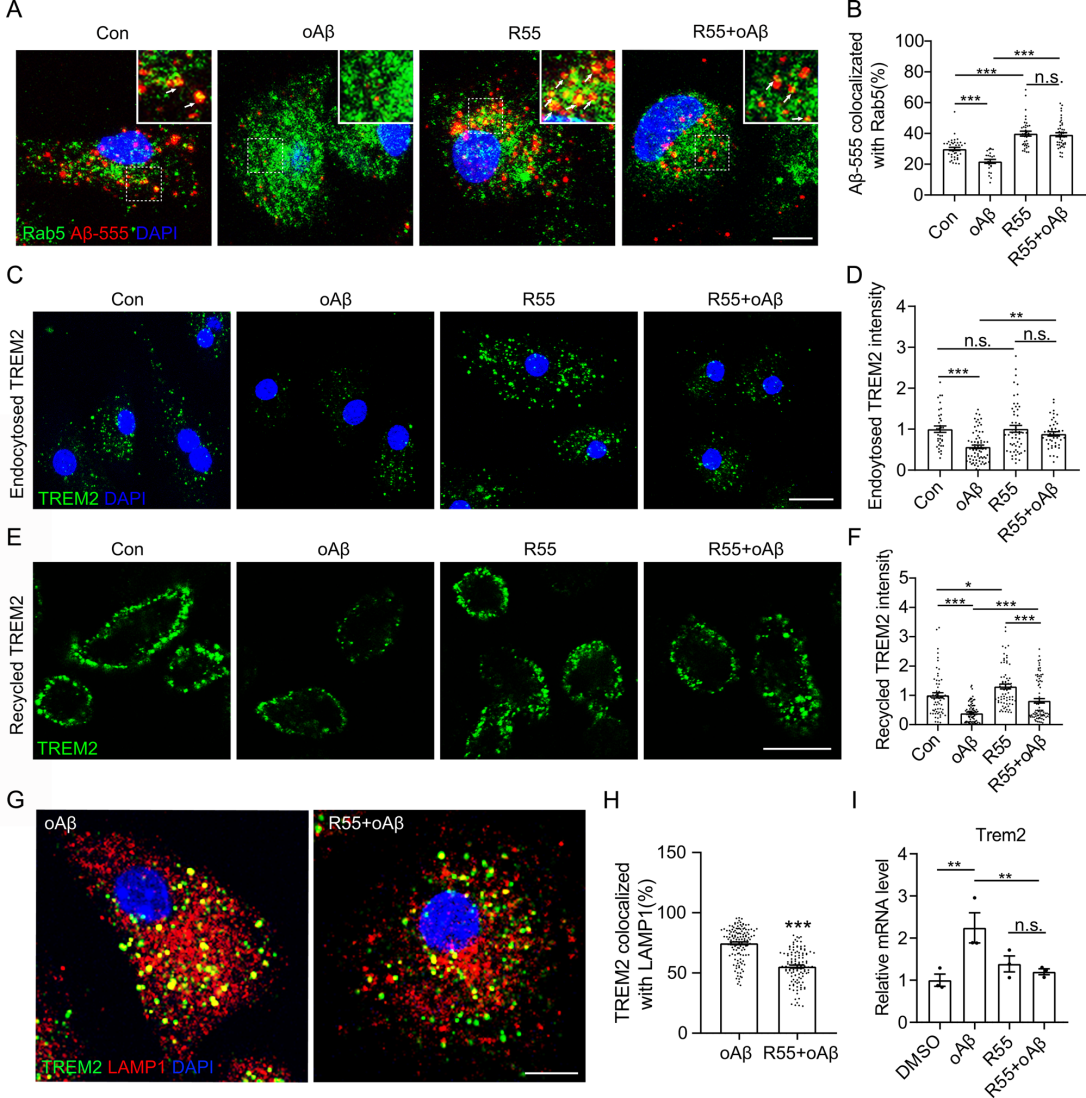
VPS35 chaperone R55 augments TREM2 recycling and Aβ endocytosis by Aβ-exposed microglia. Primary microglia were co-treated with oAβ and R55. (**A**) After R55 treatment, Aβ-exposed microglia were incubated with Aβ-555 for 30 min. The cells were then fixed and probed with anti-Rab5 antibodies, followed by counterstaining using DAPI. Scale bar = 10 μm. (**B**) Colocalization ratio of Aβ-555 with Rab5 in single cells via Manders’ colocalization coefficients. (**C**) After R55 treatment, the TREM2 internalization assay was performed in Aβ-exposed microglia. Subsequently, the cells were fixed and counterstained with DAPI. Scale bar = 20 μm. (**D**) TREM2 intensity in the Aβ-exposed microglia in panel **C** (*n* > 50). (**E**) After R55 treatment, the TREM2 recycling assay was conducted in Aβ-exposed microglia. The cells were further fixed and counterstained with DAPI. Scale bar = 20 μm. (**F**) TREM2 intensity in the Aβ-exposed microglia in panel **E** (*n* > 80). (**G**) After R55 treatment, membrane TREM2 in Aβ-exposed microglia was labeled with anti-TREM2 antibodies and allowed to undergo internalization for 60 min. The cells were then fixed and probed with anti-LAMP1 antibodies. Scale bar = 10 μm. (**H**) Colocalization ratio of TREM2 with LAMP1 in single cells via Manders’ colocalization coefficients (*n* > 100). (**I**) *Vps35* expression in R55-treated Aβ-exposed microglia was estimated via qRT-PCR (*n* = 3). *p < 0.05, **p < 0.01, and ***p < 0.001 by Student’s *t*-test (**H**) or two-way ANOVA (**B**, **D**, **F** and **I**). n.s. indicates no significant difference

Next, we explored the regulatory role of VPS35 in Aβ endocytosis in microglia by incubating Aβ-exposed microglia with R55, a molecular chaperone of VPS35 [53] . Our results showed that R55 significantly upregulated Aβ-555 endocytosis by Aβ-exposed microglia (Supp. Figure 4). Additionally, the colocalization of Aβ-555 with the early endosomal marker Rab5 was significantly upregulated after the R55 treatment of Aβ-exposed microglia (Fig. 4A and B), implying that VPS35-triggered Aβ uptake was achieved via the enhancement of Aβ endocytosis. The R55 treatment of Aβ-exposed microglia also significantly upregulated the internalization and intracellular recycling of TREM2 (Fig. 4C to F) as well as attenuated the Aβ-induced aberrant localization of TREM2 (Fig. 4G and H, Supp. Figure 5). Correspondingly, R55 treatment diminished the Aβ-induced compensatory upregulation of *Trem2* transcription in Aβ-exposed microglia (Fig. 4I). All these findings demonstrated that VPS35 might be involved in Aβ-induced TREM2 dysfunction, and that upregulation of VPS35-retromer stability significantly alleviating the aberrant localization of TREM2 and augmenting TREM2-dependent Aβ endocytosis.

**Fig. 5:**
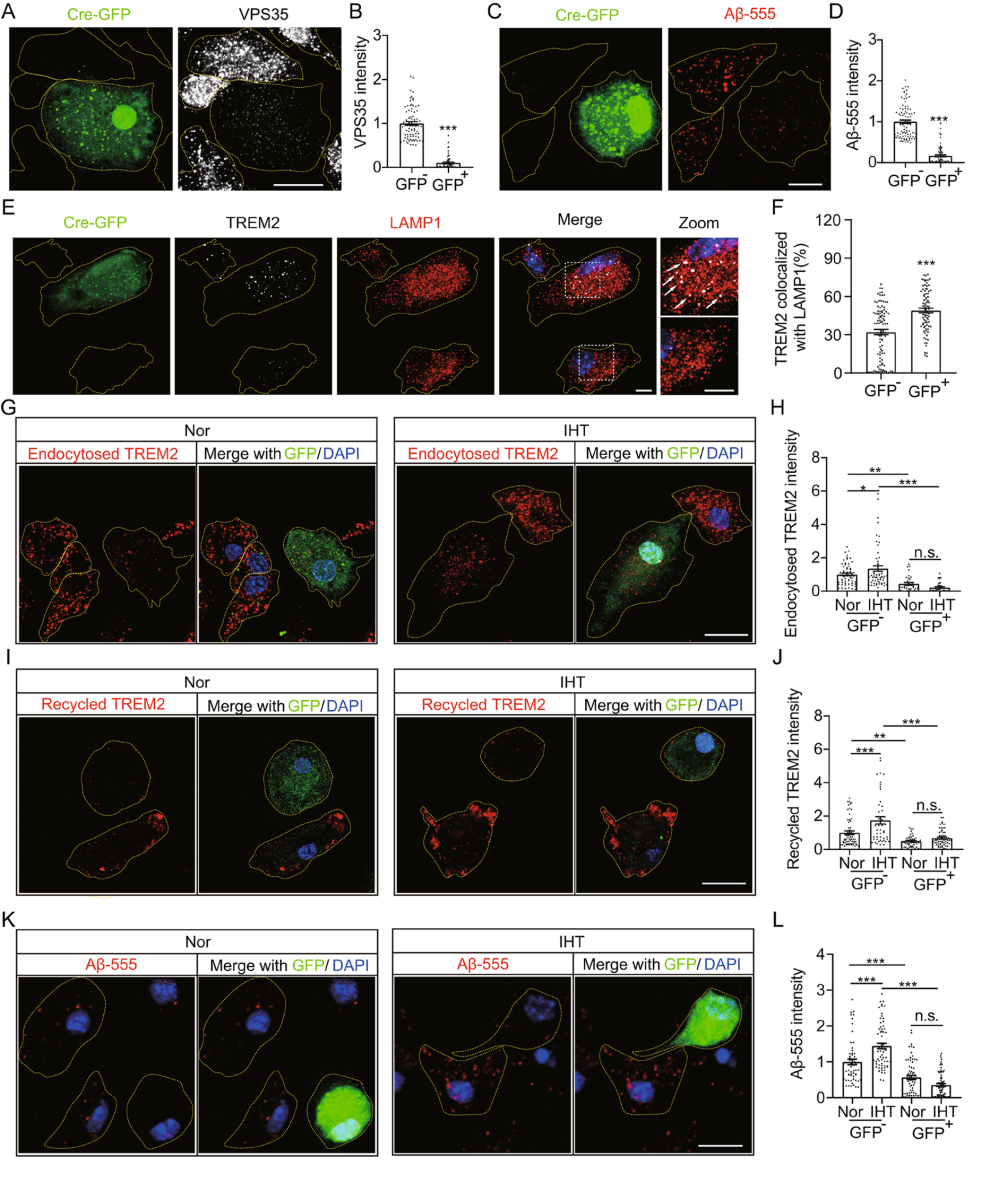
IHT-induced upregulation of Aβ endocytosis by Aβ-exposed microglia depends on VPS35. Primary microglia were transfected with lentivirus expressing Cre-GFP. (**A**) Cells were labeled with anti-VPS35 antibodies. Scale bar = 20 μm. (**B**) VPS35 intensity in GFP^−^ and GFP^+^ cells in panel **A** (*n* > 50). (**C**) Cells were incubated with Aβ-555 for 30 min and then fixed. Scale bar = 10 μm. (**D**) Aβ-555 intensity in GFP^-^ or GFP^+^ cells in panel **C** (*n* > 80). (**E**) Cells were labeled with anti-LAMP1 and anti-TREM2 antibodies, followed by counterstaining using DAPI. Scale bar = 5 μm. (**F**) Colocalization ratio of TREM2 with LAMP1 in single cells via Manders’ colocalization coefficients (*n* > 100). (**G** to **J**) Cells were treated with oAβ to construct Aβ-exposed microglia, followed by treatment with IHT. TREM2 internalization (**G**), TREM2 recycling (**I**), and Aβ-555 uptake (**K**) assays were conducted in the Aβ-exposed microglia (Scale bar = 20 μm). Endocytosed TREM2 (**H**), recycled TREM2 (**J**), and internalized Aβ-555 (**L**) were quantified from panels **G, I**, and **K**, respectively (*n* > 50). *p < 0.05, **p < 0.01, and ***p < 0.001 by Student’s *t*-test (**B**, **D** and **F**) or two-way ANOVA (**H**, **J** and **L**). n.s. indicates no significant difference. Nor, normoxia; IHT, intermittent hypoxia training

Considering that IHT upregulated VPS35 in DAM, we hypothesized that IHT elevated the Aβ uptake by DAM via VPS35 upregulation. We further tested this hypothesis by knocking out *Vps35* using lentiviruses expressing Cre-GFP fusion proteins to infect VPS35^fl/fl^ microglia. As illustrated in Fig. 5A and B, VPS35 was significantly reduced in GFP^+^ microglia. In support of our hypothesis, Aβ-555 endocytosis was significantly reduced in GFP^+^ microglia (Fig. 5C and D), suggesting that Aβ-555 endocytosis by microglia depended on VPS35. Moreover, the colocalization of TREM2 with LAMP1 significantly increased in VPS35-deficient microglia (Fig. 5E and F), suggesting that the capacity of TREM2 endocytosis and recycling depended on VPS35. Furthermore, we observed that IHT did not exhibit an ameliorating effect on the intracellular transport of TREM2 (Fig. 5G to J) and Aβ-555 internalization (Fig. 5K and L) in VPS35-deficient Aβ-exposed microglia. These results highlighted that the upregulation of TREM2-mediated Aβ endocytosis by IHT in Aβ-exposed microglia depended on VPS35.

### IHT-induced attenuation of AD pathology depends on VPS35

**Fig. 6:**
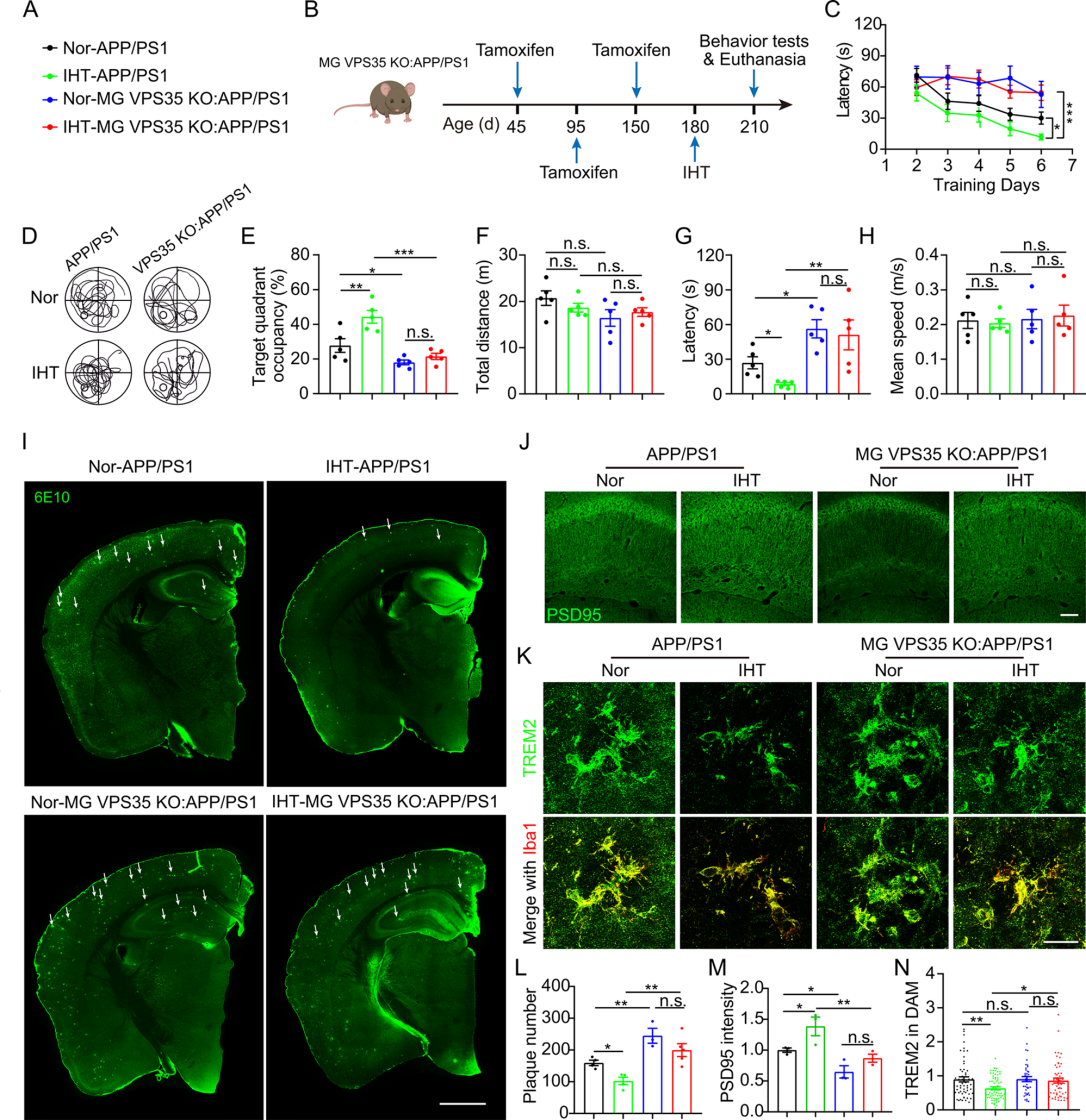
IHT exhibits no significant improvement in Aβ pathology in 7-months microglial VPS35-deficient APP/PS1 mice. (**A**) Labeling information for different groups. (**B**) Flowchart of the development of IHT-treated MG VPS35 KO: APP/PS1 mice. (**C**) Escape latency of the IHT-treated MG VPS35 KO: APP/PS1 mice to find the platform for the first during the MWM training period. (**D**) Swimming trajectories of the mice in the MWM probe period. (**E**) The dwelling time in the target quadrant was calculated as the percentage of the total time in the MWM probe test. (**F**) Total moving distance in the target quadrant was calculated in the MWM probe test. (**G**) The escape latency of mice to archive the target quadrant during the MWM probe period. (**H**) The average swimming speed of mice during the MWM probe test (*n* = 5). (**I**) Brain sections of the IHT-treated MG VPS35 KO: APP/PS1 mice were labeled with 6E10 antibodies. White arrows indicate plaques. Scale bar = 2 mm. (**J)** Brain sections of the IHT-treated MG VPS35 KO: APP/PS1 mice were probed with anti-PSD95 antibodies to label synapses in the CA1 region. Scale bar = 150 μm. (**K**) Brain sections of the IHT-treated MG VPS35 KO: APP/PS1 mice were stained with anti-Iba1 and anti-TREM2 antibodies. Scale bar = 20 μm. (**L**) Number of Aβ plaques in the hippocampus and cortex regions in panel **I**. (**M**) PSD95 intensity in the CA1 region in panel **J**. (**N**) TREM2 intensity in the DAM of CA1 region in panel **K** (five mice in each group, with five to eight cells per mouse). *p < 0.05, **p < 0.01, and ***p < 0.001 by two-way ANOVA. n.s. indicates no significant difference. Nor, normoxia; IHT, intermittent hypoxia training

We further examined whether IHT ameliorated AD pathology via microglial VPS35 by specifically knocking out VPS35 in microglia in the background of AD mice (Fig. 6A, Supp. Figure 6). Our findings showed that the cognition of MG VPS35 KO: APP/PS1 mice were significantly lower than that of VPS35 ^fl/fl^:TG mice, exhibiting no significant improvement even after IHT (Fig. 6C to H). Correspondingly, IHT did not demonstrate an inhibitory effect on the Aβ accumulation in the brains of MG VPS35 KO: APP/PS1 mice (Fig. 6I and L), along with no significant alleviation in neuronal damage (Fig. 6J and M). Furthermore, we investigated whether the IHT-induced improvement in TREM2 function depended on VPS35 (Fig. 6K). Our results indicated that IHT did not reduce TREM2 in the AD mice with VPS35-deficient DAM (Fig. 6N). Based on these findings, the IHT-associated enhancement of Aβ endocytosis and clearance by DAM was dependent on microglial VPS35.

### TFEB transcriptionally regulates VPS35 expression by binding to the promoter region of Vps35 in murine microglia

**Fig. 7:**
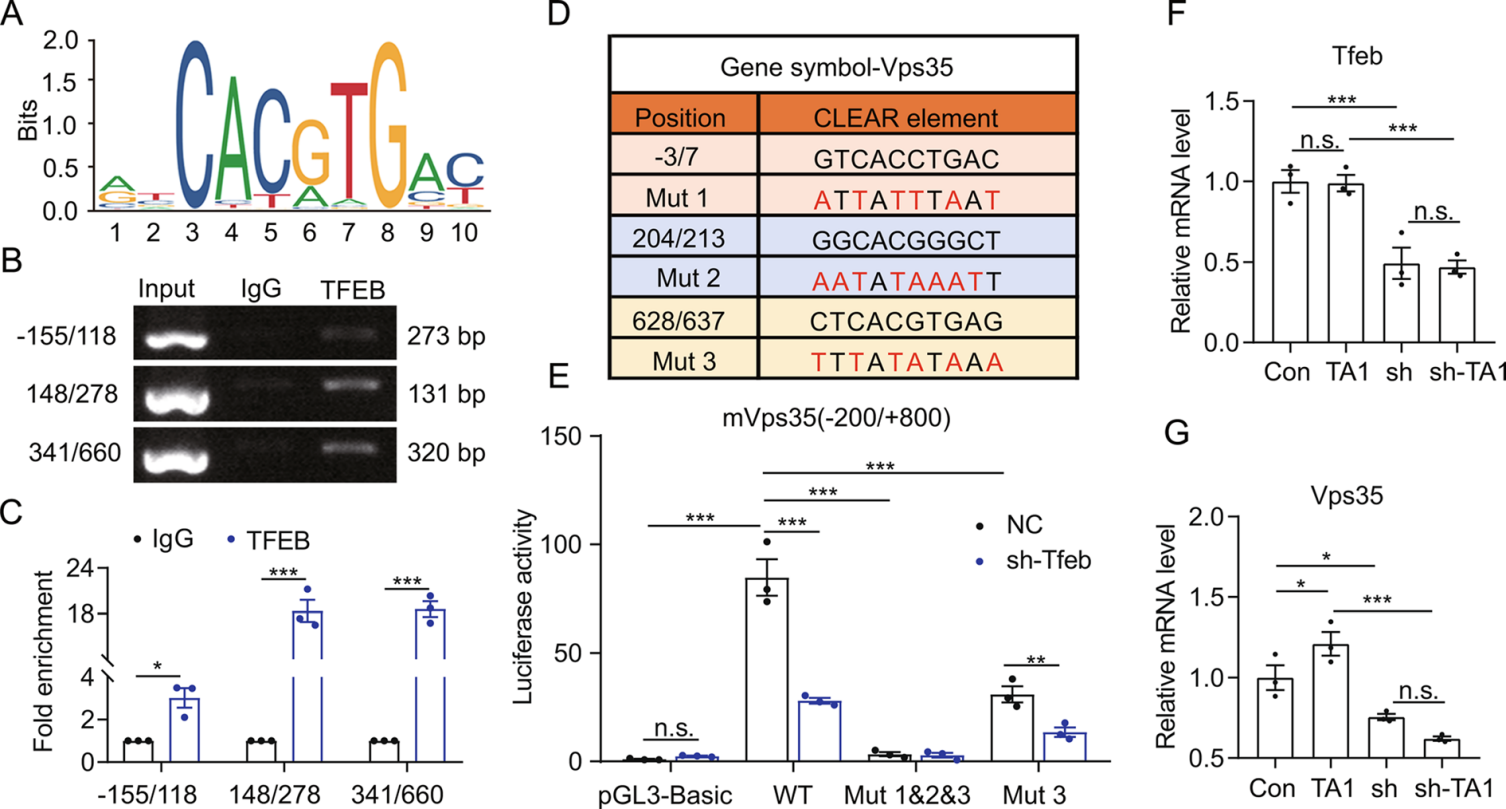
TFEB transcriptionally regulates Vps35 in BV2 cells. (**A**) A consensus TFEB-binding motif within the ChIP-seq peaks. (**B**) ChIP-PCR was performed to identify potential TFEB-binding elements in the *Vps35* gene regulatory regions. IgG was used as a negative control. (**C**) ChIP-qPCR was conducted to detect the fold enrichment of the three identified CLEAR elements. (**D**) Sequences of ChIP-based CLEAR elements and mutants. (**E**) Dual luciferase activities of the constructs containing the promoter region of *Vps35* with either intact (WT) or mutated (Mut) CLEAR elements were compared between shControl (NC) and sh*TFEB* HEK293T cells. (**F** and **G**) *Tfeb* and *Vps35* expressions in TA1-treated sh*Tfeb* BV2 cells were estimated with qRT-PCR. *n* = 3, *p < 0.05 and ***p < 0.001 by Student’s *t*-test (**C**) or two-way ANOVA (**E** to **G**). n.s. indicates no significant difference

A previous study by Rachel et al. reported that TFEB transcriptionally regulates human *VPS35* [41]. According to this finding, we hypothesized that VPS35 upregulation by IHT was related to TFEB. Therefore, we explored the regulatory function of TFEB on mouse VPS35 to substantiate this hypothesis. As presented in Fig. 7A to C, TFEB binds to three coordinated lysosomal expression and regulation (CLEAR) elements near the promoter region of *Vps35*. Subsequently, we investigated the transcriptional activation of *Vps35* by TFEB. Reporter systems were first constructed for monitoring *Vps35* transcription (Fig. 7D), and TFEB silencing was found to significantly repress *Vps35* transcription (Fig. 7E). Next, we observed that mutating the three CLEAR elements caused TFEB to lose its transcriptional regulation activity on *Vps35*, suggesting that TFEB regulated *Vps35* transcription via the three CLEAR elements. Consistent with the above findings, VPS35 expression in BV2 cells was significantly downregulated after the silencing of *Tfeb* (Fig. 7F and G). In contrast, VPS35 was significantly upregulated after administering TA1, an agonist of TFEB [43], while TA1 did not exhibit its regulatory effect on sh*Tfeb* BV2 cells. All these results indicated that TFEB directly regulated VPS35.

### IHT ameliorates Aβ endocytosis by DAM and attenuates Aβ pathology by upregulating TFEB-regulated VPS35

**Fig. 8:**
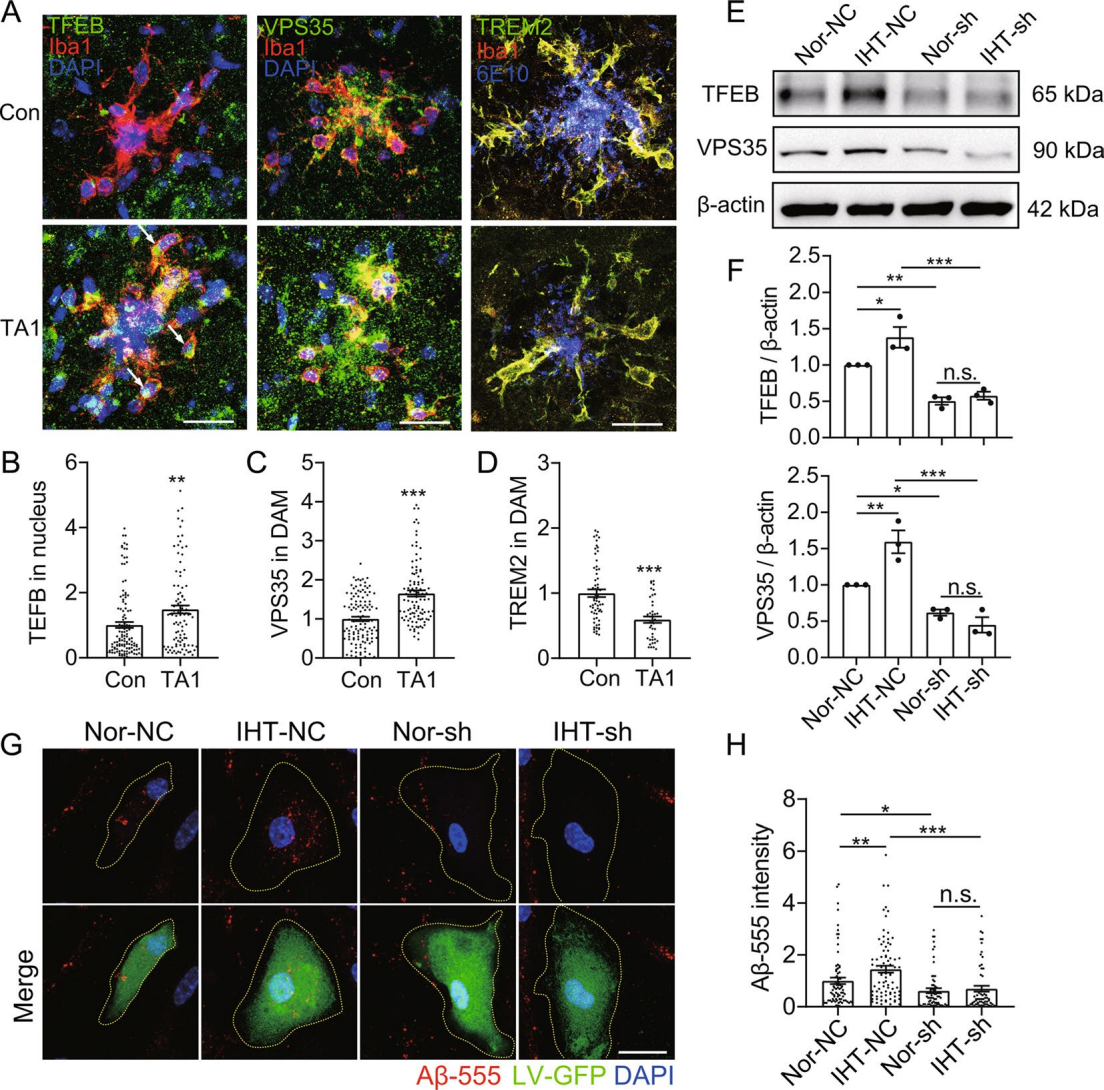
IHT-induced attenuation of Aβ pathology and amelioration of Aβ endocytosis by DAM depends on TFEB. 9-month-old APP/PS1 mice were treated with TA1. (**A**) Brain sections of TA1-treated APP/PS1 mice were stained with anti-TFEB/anti-VPS35/anti-TREM2 and anti-Iba1 antibodies, and microscopy images of DAM in CA1 region were captured. Scale bar = 20 μm. (**B** to **D**) TFEB (**B**), VPS35 (**C**), and TREM2 (**D**) intensities in the DAM in panel **A** (six mice in each group, with 10 cells per mouse). (**E**) Sh*Tfeb* BV2 cells were used to construct Aβ-exposed microglia and then treated with IHT. TFEB and VPS35 expression levels were detected via Western blotting. (**F**) Grayscale values of the protein bands in panel **E** (*n* = 3). (**G**) After treatment with IHT, sh*Tfeb* Aβ-exposed microglia were incubated with Aβ-555 for 30 min, followed by fixation and counterstaining with DAPI. Scale bar = 10 μm. (**H**) Aβ-555 intensity in the GFP^+^ cells in panel **G** (*n* > 50). *p < 0.05, **p < 0.01, and ***p < 0.001 by Student’s *t*-test (**B** to **D**) or two-way ANOVA (**F**, **H**). n.s. indicates no significant difference. Nor, normoxia; IHT, intermittent hypoxia training

We have reported that IHT effectively upregulated nuclear TFEB in Aβ-exposed microglia, and TA1 treatment significantly attenuated Aβ pathology in the brain, including a reduction in the number of plaques and decreased Aβ load [38]. Based on our finds in the current work, we suspected that upregulation of VPS35 by IHT might be linked to TFEB activation. Indeed, IHT led to a significant upregulation in TFEB and VPS35 as well as a significant decline in TREM2 in DAM (Fig. 8A to D), implying an important regulatory role of the TFEB–VPS35–TREM2 axis in DAM function. Moreover, IHT could not retain this upregulated VPS35 expression after the silencing of TFEB in Aβ-exposed BV2 (Fig. 8E and F, Supp. Figure 7), emphasizing that IHT-promoted VPS35 expression was dependent on TFEB. Subsequently, we observed that IHT did not have a promotional effect on Aβ endocytosis by Aβ-exposed microglia when silencing of TFEB (Fig. 8G and H). These findings demonstrated that IHT-associated improvement in Aβ endocytosis by DAM depended on TFEB-regulated VPS35.

**Fig. 9:**
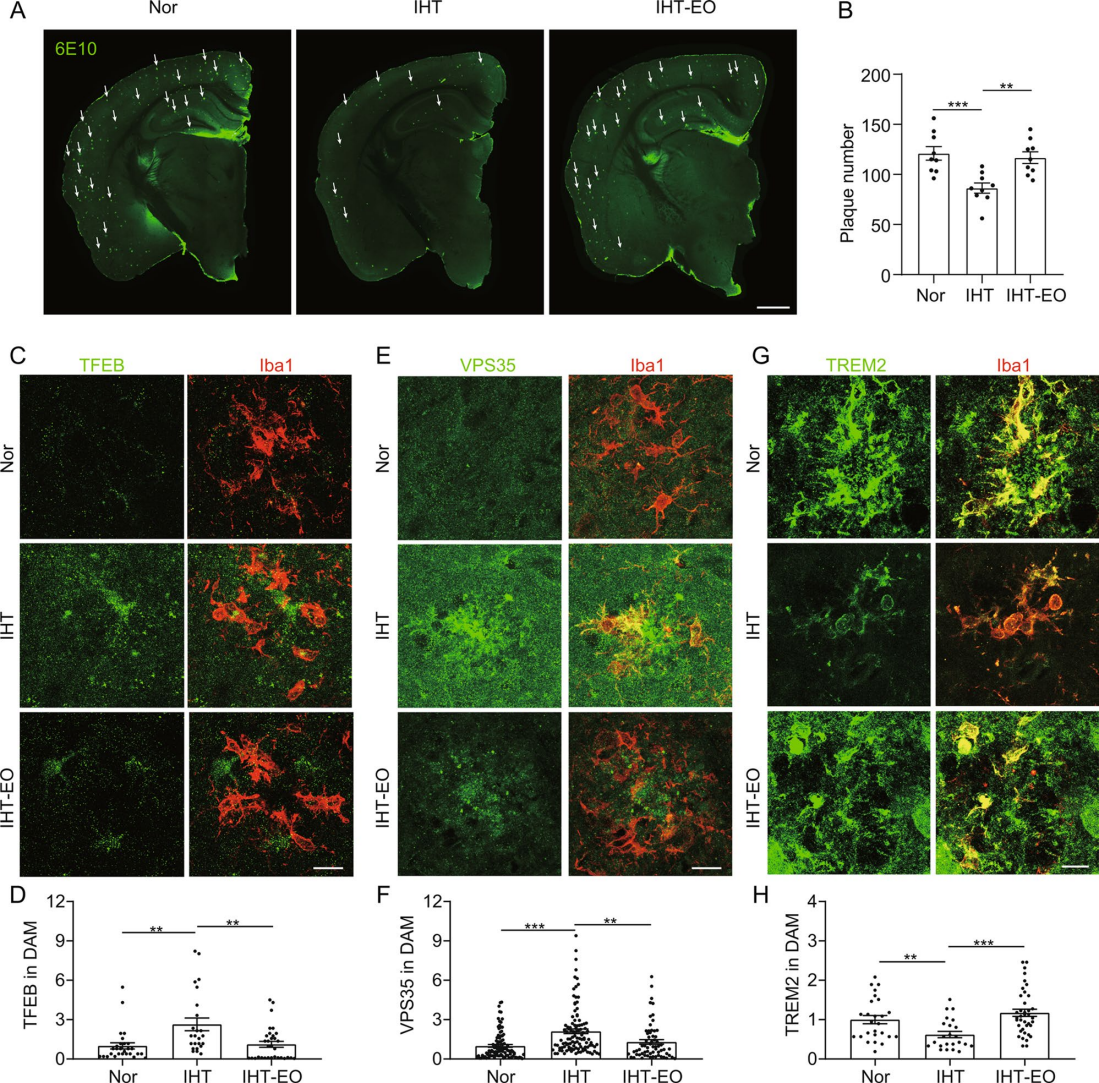
TFEB inhibition reduces the IHT-induced upregulation of VPS35 in DAM 9-months APP/PS1 mice brains. (**A**) Brain sections of APP/PS1 mice treated with IHT or EO were probed with 6E10 antibodies, and microscopy images of the Aβ plaques were acquired. White arrows indicate plaques. Scale bar = 2 mm. (**B**) Number of Aβ plaques in the hippocampus in panel **A** (*n* = 9). (**C** to **H**) Brain sections of IHT or EO-treated APP/PS1 mice were stained with anti-TFEB (**C**), anti-VPS35 (**E**), or anti-TREM2 (**G**) and anti-Iba1 antibodies, and microscopy images of DAM in CA1 region were captured. Scale bar = 20 μm. TFEB (**D**), VPS35 (**F**), and TREM2 (**H**) intensities in the DAM cells in panels **C, E**, and **G**, respectively. *p < 0.05, **p < 0.01, and ***p < 0.001 by Student’s *t*-test (**D**, **F** and **H**: four mice in each group, with three to five cells per mouse). Nor, normoxia; IHT, intermittent hypoxia training

Additionally, we found that IHT in microglial VPS35-deficient AD mice did not exhibit an ameliorative effect on AD pathology, whereas the nuclear expression of TFEB in DAM remained elevated (Supp. Figure 8). Thus, TFEB may have a regulatory role upstream of VPS35 in AD pathology. Further, treatment with EO, a TFEB inhibitor, significantly suppressed the IHT-enhanced Aβ loading in AD mice brains (Fig. 9A and B), along with a significant reduction in nuclear TFEB expression in DAM (Fig. 9C and D). EO treatment also reversed the alleviating effect of IHT on VPS35 and TREM2 expression in DAM (Fig. 9E to H). Therefore, these results indicated that IHT-induced improvement of the VPS35–TREM2 axis in DAM was dependent on TFEB.

## Discussion

IHT has been previously demonstrated to ameliorate AD pathology in numerous ways. For example, Myoung-Gwi et al. found that IHT in 12-month-old 3xTg-AD mice effectively upregulated erythropoietin and brain-derived neurotrophic factor in the brain, reduced the brain Aβ load, and improved spatial learning and memory abilities [35]. Another study by Xiangpei Yue et al. showed that IHT lowered the levels of pro-inflammatory cytokines IL-1β and IL-6 in APP/PS1 mice brains and diminished the expression of AD pathology-related genes, thus reducing β-secretase-mediated cleavage of APP and preventing Aβ overloading in the brain [54]. IHT was also reported to convert resting microglia to anti-inflammatory microglia and effectively enhance microglial phagocytosis [55]. Microglia are involved in cognitive regulation by participating in neuronal circuit formation and development, modulating synaptic plasticity to influence axon growth and neuronal localization [56]. In addition, microglia damage neurons and affect cognitive function by releasing inflammatory cytokines and reducing neurotrophic factors [57]. Thus, targeting abnormally activated microglia facilitates the recovery of cognitive functions. Our previous research demonstrated that IHT effectively upregulated the nuclear translocation of TFEB in DAM via the AKT–MAPK–mTOR pathway and improved the autophagic lysosomal function of DAM, ultimately minimizing Aβ load in APP/PS1 mice brains [38]. In the present study, we revealed that IHT effectively enhanced Aβ internalization by DAM. Considering that Aβ internalization is the initial step in its clearance process, our study findings offer valuable insight into refining the mechanism of IHT to further alleviate Aβ pathology and provide basic data supporting the clinical application of IHT in AD treatment.

We have demonstrated a positive effect of IHT on Aβ clearance [38]., the critical regulatory role of LC3-related endocytosis in Aβ clearance [16] led us to consider whether IHT could modulate Aβ uptake. Indeed, it has been reported that down-regulation or knockdown of TREM2 results in Aβ accumulation [50, 58]. Therefore, the observed downregulation of TREM2 in disease-associated microglia (DAM) following the introduction of IHT was of great interest to us. Given the significant regulatory role of intracellular recycling of TREM2 in Aβ uptake, we demonstrated that IHT enhances the membrane expression of TREM2, thereby contributing to the endocytosis of Aβ by DAM. TREM2 is a microglial membrane receptor that regulates phagocytosis, apoptosis, and inflammation as well as responses to Aβ [59]. TREM2 modulates cognitive function in AD mice by mediating Aβ endocytosis and clearance by DAM [17]. Zhang et al. found that exogenous administration of soluble TREM2 ameliorated cognitive deficits in AD mice through activation of transgelin-2 ^60^. Ruganzu et al. reported that overexpression of TREM2 improved cognitive function by inhibiting neuroinflammation through the JAK/STAT/SOCS pathway [60, 61]. Conversely, TREM2 deficiency inhibits DAM, which in turn reduces the microglial response to Aβ and diminishes Aβ clearance [62]. Previous research has also highlighted that TREM2^R47H^ mutation significantly reduces Aβ binding and exacerbates Aβ deposition in the brain [63], while TREM2^H157Y^ mutation induces M1-type microglia, increases the release of IL-1β, IL-6, and TNFα, and exacerbates AD pathology by decreasing membrane TREM2 levels [64]. Although TREM2 upregulation favors Aβ clearance in the brain, elevated TREM2 levels in the brains of patients with AD suggest that TREM2 function rather than its concentration has a more critical role in Aβ homeostasis [65]. In early AD, the compensatory upregulation of TREM2 in DAM was reported to enhance the Aβ clearance ability of DAM. However, the downregulation of the transporter protein, VPS35, resulted in the compensatory upregulation of TREM2 being unable to counteract the sustained decrease in the endocytosis capacity of DAM. In the current study, transcription of TREM2 in DAM was significantly downregulated after IHT, but Aβ endocytosis by DAM was significantly increased. All these findings indicated that focusing on TREM2 function rather than its expression levels might be more helpful in the context of AD pathology. The restoration of TREM2 membrane expression may have attenuated the compensatory upregulation of TREM2 in DAM.

Additionally, our study showed that IHT-induced improvement in TREM2 function in DAM depended on the VPS35 transporter protein. VPS35 is a core subunit of the retromer complex, which regulates the endocytosis, autophagy, and lysosomal degradation pathways by translocating endocytosed cargos to the plasma membrane [66]. VPS35 deficiency has been previously demonstrated to induce microglial polarization to the pro-inflammatory type [42]. VPS35 deficiency was also found to severely impede Aβ endocytosis by DAM and exacerbate cognitive deficits in 5xFAD mice [25]. Based on the downregulation of VPS35 in the brains of patients with AD and AD model mice, we hypothesized that VPS35 downregulation in DAM exacerbated AD progression. In contrast, VPS35 upregulation in DAM may mitigate AD progression by improving the inflammatory response and Aβ internalization in DAM. VPS35 has also been suggested to play a crucial regulatory role in neurons [67]. Moreover, VPS35 defects can lead to abnormal APP sorting to the lysosome, resulting in excess production of Aβ monomers and elevated brain Aβ load [68]. In this context, R55, a molecular chaperone of VPS35, was found to reduce incorrectly trafficked APP and Aβ generation by improving retromer stability [53]. We also observed a significant enhancement of the background of VPS35 after IHT treatment. Since VPS35 is highly expressed in both neurons and microglia, we hypothesize that there is a high probability that IHT upregulated VPS35 in both neurons and microglia. During the development of AD, VPS35 participates in the recycling of neuronal APP and promotes the detachment of APP from endosomes to avoid lysosomal metabolism and thus the production of excess Aβ [34, 68]. Therefore, we hypothesized that IHT could attenuate the lysosomal degradation of APP by upregulating VPS35 in neurons, thereby decreasing the production of Aβ monomers. Yue et al. reported that IHT was able to effectively downregulate the expression level of BACE1 in neurons of APP/PS1 mice, and reduce the production of Aβ by neurons [69]. Based on the broad regulatory effects of IHT, it is possible that IHT plays an active role in Aβ production and clearance through multiple pathways. VPS35 is also involved in various neurodegenerative diseases, such as Parkinson’s disease (PD) and lateral sclerosis of the spinal cord. IHT effectively upregulated dopamine release from peripheral chemoreceptors in the carotid arteries of patients with PD, consequently exhibiting a protective role in PD prevention and treatment [70]. Based on these vital functions of VPS35 in the nervous system, IHT-induced modulation of VPS35 has a potential therapeutic role in numerous neurological diseases.

In addition to TREM2, microglia endocytose Aβ via various Scavenger receptors, including CD36 [71], formyl peptide receptor [72], and lipoprotein receptor [73]. The Retromer complex, which includes VPS35, mediates the intracellular recycling and reutilization of these receptors [74]. Previous studies have shown that the Retromer enhances Aβ uptake and reduces Aβ plaque formation in the brains of APP/PS1 mice by increasing the cycling efficiency of CD36 and TREM2 [75]. It also reduces the cleavage of APP to generate Aβ by facilitating the return of SORLA to the membrane [76]. In fact, the VPS26/Retromer also facilitates the sorting of SORLA and APP by recycling them to the Golgi [77]. Based on these findings, we hypothesize that IHT may have the potential to improve Aβ pathology by enhancing the intracellular recycling of these and other receptors through the upregulation of VPS35 in disease-associated microglia (DAM).

Our results also demonstrated that IHT upregulated VPS35 by activating its transcription activator, TFEB. Rachel Curnock et al. showed that TFEB transcriptionally activates VPS35 in HeLa cells [41], corroborating that VPS35 is a target gene of TFEB. Prior studies have also found that TFEB is strongly associated with AD, wherein TFEB is downregulated in the brains of patients with AD and AD model mice as well as linked to reduced autophagy [78]. Accordingly, TFEB upregulation is reported to effectively attenuate Aβ deposition in the brain and ameliorate AD pathology by upregulating autophagy [43], indicating that endocytosis-autophagy are inextricably linked to overall cellular functions. It has been reported that endocytosis regulates subsequent autophagy, and LC3-associated endocytosis facilitates autophagic clearance of Aβ and attenuates the rapid progression of AD [16]. Regulation of endocytosis-associated membrane receptors are also involved in the regulation of autophagy function. Li et al. found that the absence of VPS35 significantly down-regulates LC3 and autophagic flux [60]. A study by Israel C Nnah et al. highlighted that TFEB, a key factor in autophagy regulation, promotes the transcription of endocytosis-related genes [79]. Our study also confirmed that TFEB improved TREM2 intracellular recycling by enhancing VPS35 expression, supporting the regulatory role of TFEB in the endocytosis process. Therefore, the present study broadens our understanding of the mechanism of TFEB-modulated Aβ endocytosis by microglia and strengthens the evidence of the relationship between the endocytosis and autophagy processes in microglia. Previous research has suggested that Aβ clearance by DAM depends on LANDO [16]. Our study findings indicate that LANDO may be associated with TFEB-regulated VPS35. Moreover, TFEB induces the downregulation of endocytosis by DAM via VPS35, while VPS35 is also involved in cellular autophagy regulation [60]. All these observations imply that the endocytosis–autophagy pathway may be inextricably linked to cellular function, with problems in either of their components disrupting cell fate. Thus, phagocytosis-targeted treatment under pathological conditions should be focused on the endocytosis–autophagy pathway rather than directed toward only one aspect.

## Conclusions

In conclusion, our study revealed that IHT reduced Aβ load in the brain by improving the endocytosis activity of DAM. Additionally, we elucidated the regulatory mechanism of the TFEB–VPS35–TREM2 axis in Aβ endocytosis by DAM. Our findings demonstrated that IHT upregulated VPS35 transcription by activating the nuclear translocation of TFEB in DAM, leading to enhanced VPS35-dependent recycling and membrane expression of TREM2 and increased Aβ endocytosis by DAM. These alterations ultimately contributed to reducing brain Aβ load and cognitive impairment. Our study highlights that targeting VPS35 has an ameliorative effect on Aβ endocytosis and pathology and provides further evidence supporting the application of IHT for treating neurodegenerative diseases, including AD.

### Electronic supplementary material

Below is the link to the electronic supplementary material.

Supplementary Material 1

Supplementary Material 2

## Data Availability

The authors confirm that the data supporting the findings of this study are available within the article and its Supplementary material. Raw data that support the findings of this study are available from the corresponding author, upon reasonable request.

## Abbreviations

Aβ: Beta-amyloid
AD: Alzheimer’s disease
APP: Amyloid precursor protein
HiLyte^TM^: Fluor 555 labeled oligomer Aβ1–42
CLEAR: Coordinated Lysosomal Expression and Regulation
IHT: Intermittent hypoxia training
LANDO: LC3-associated endocytosis
MWM: Morris water maze
oAβ: Aβ oligomer
DAM: Disease-associated microglia
SNXs: Sorting nexins
SORLA: Sortilin-related receptor
TFEB: Transcription factor EB
TA1: TFEB activator 1
TFR: Transferrin receptor 1
TG: APP/PS1 transgenic mouse
TREM2: Triggering receptor expressed on myeloid cells 2
VPS35: Vacuolar protein sorting 35
WT: Wide type
