# Supplementary material for: Intermittent hypoxia training enhances Aβ endocytosis by plaque associated microglia via VPS35-dependent TREM2 recycling in murine Alzheimer’s disease
Source: Alzheimers Res Ther. 2024 Jun 3;16:121. doi: 10.1186/s13195-024-01489-6 (PMC11145795; doi:10.1186/s13195-024-01489-6)
Supplement: Supplementary file 1 — Supplementary Material 1 [file 13195_2024_1489_MOESM1_ESM.docx]

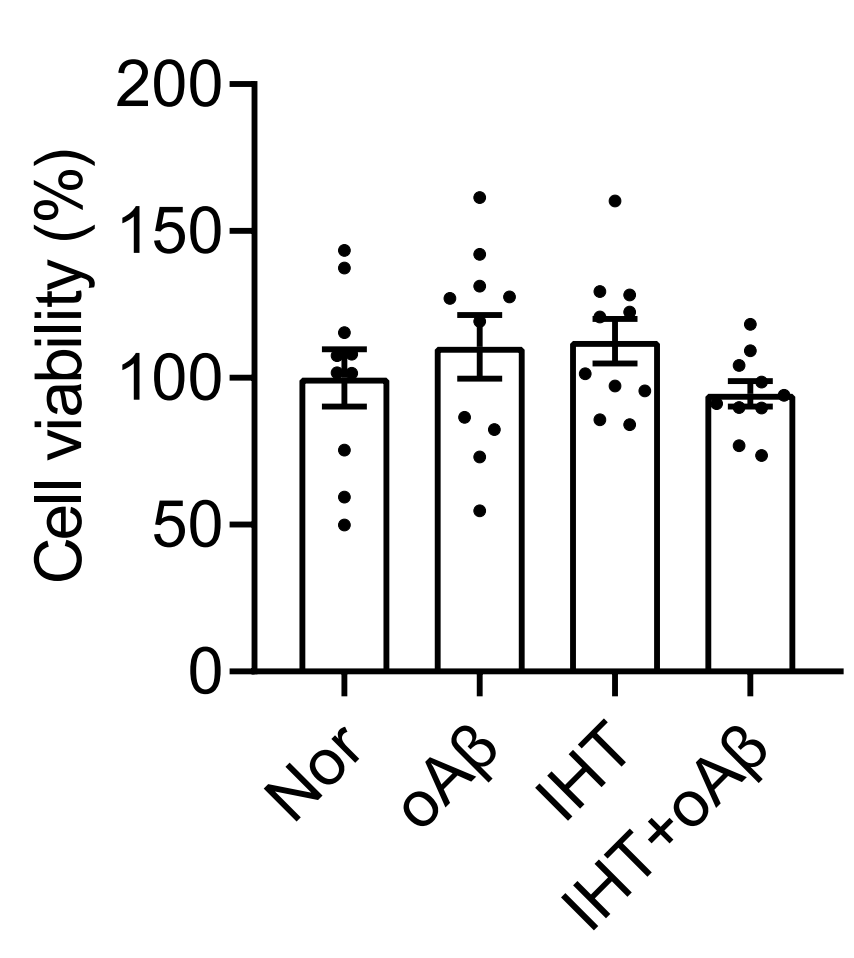


**Supp Fig. 1 IHT has no significant effect on cell** **viability.** Primary microglia were treated with oAβ for 12 h. Cell viability was tested by CCK-8. n = 10, n.s. indicated no significant difference by two-way ANOVA. Nor, normoxia.


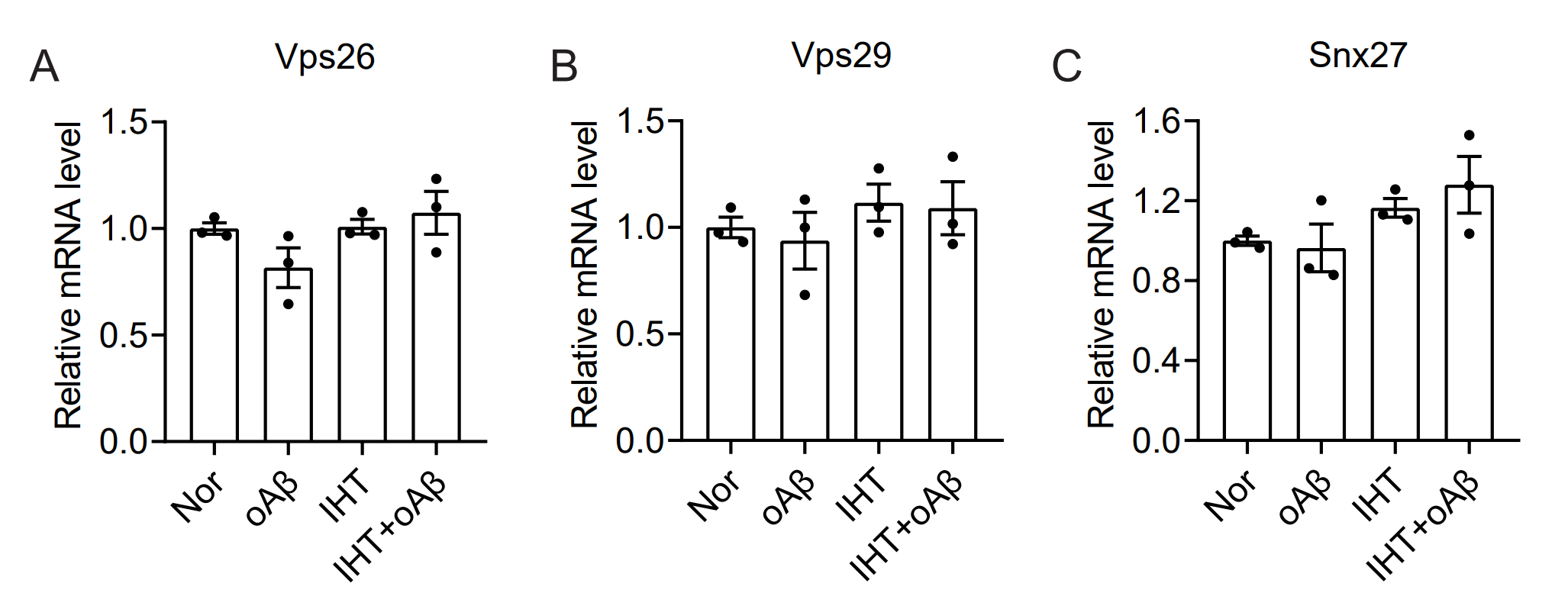


**Supp Fig. 2 IHT has no significant effect on** ***Vps26*, *Vps29* and *Snx27* in Aβ-exposed microglia.** Primary microglia were treated with oAβ for 12 h. After treated with IHT, the mRNA level of *Vps26*, *Vps29* and *Snx27* were measured using qRT-PCR. n = 3, n.s. indicated no significant difference by two-way ANOVA. Nor, normoxia.


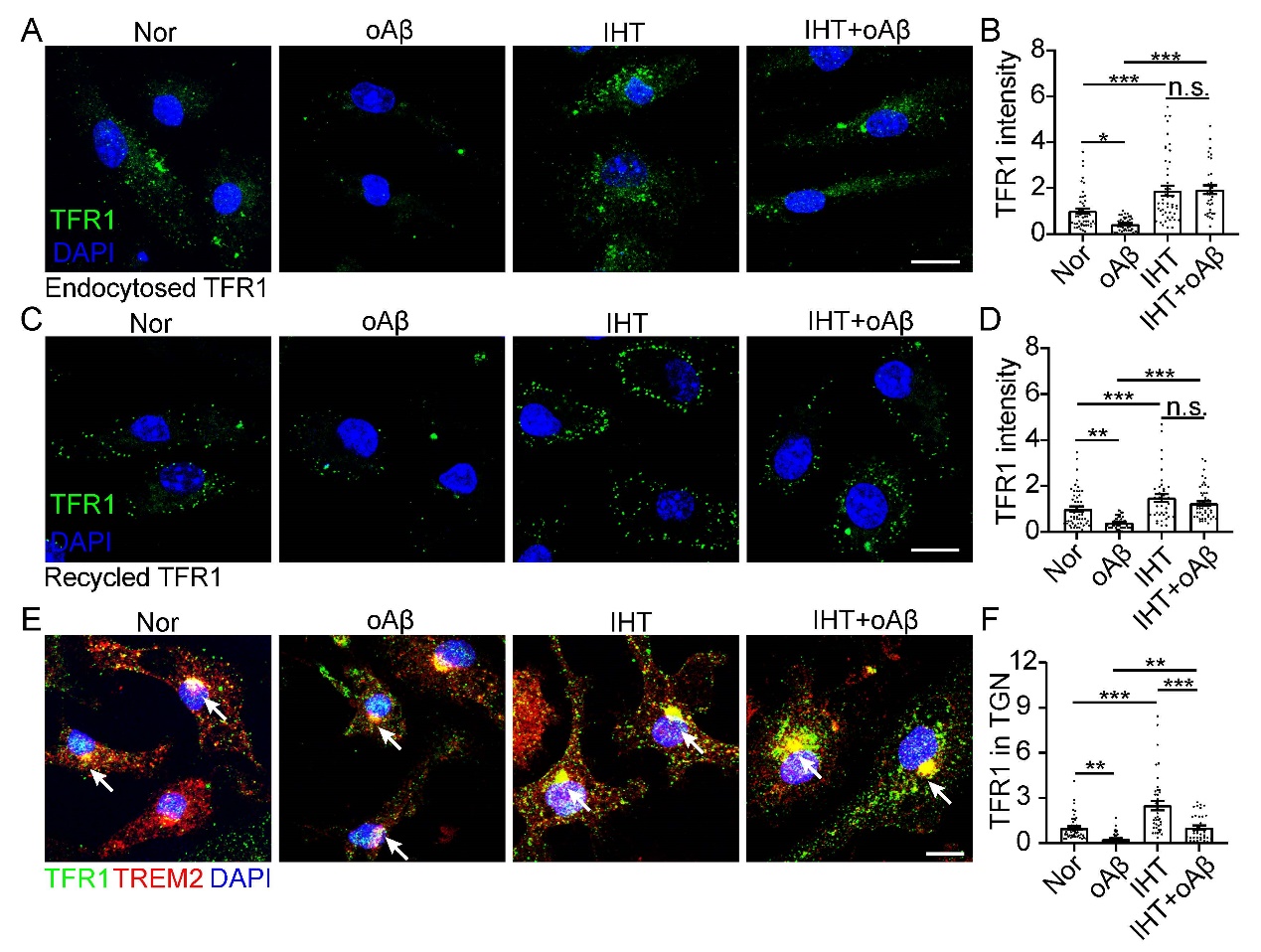


**Supp Fig. 3 IHT enhances TFR1 recycling in Aβ-exposed microglia.** Primary microglia were treated with oAβ for 12 h. (**A**) After treated with IHT, the TFR1 internalization assay was performed. The cells were then fixed and counterstained with DAPI. Scale bar = 10 μm. (**B**) TFR1 intensity in Aβ-exposed microglia in panel **A** (*n* > 40). (**C**) TFR1 recycling assay in Aβ-exposed microglia after treatment with IHT treatment. Scale bar = 10 μm. (**D**) TFR1 intensity in Aβ-exposed microglia in panel **C** (*n* > 40)**.** (**E**) Aβ-exposed microglia were fixed and co-stained with anti-TREM2 and anti-TFR1 antibodies after IHT treatment. Then cells were counterstained with DAPI. The arrows indicate the location of the trans Golgi network (TGN). Scale bar = 10 μm. (**F**) TFR1 intensity in Aβ exposed microglia in panel **E** (*n* > 40). * *p* < 0.05, ** *p* < 0.01 and *** *p* < 0.001 by two-way ANOVA. Nor, normoxia.


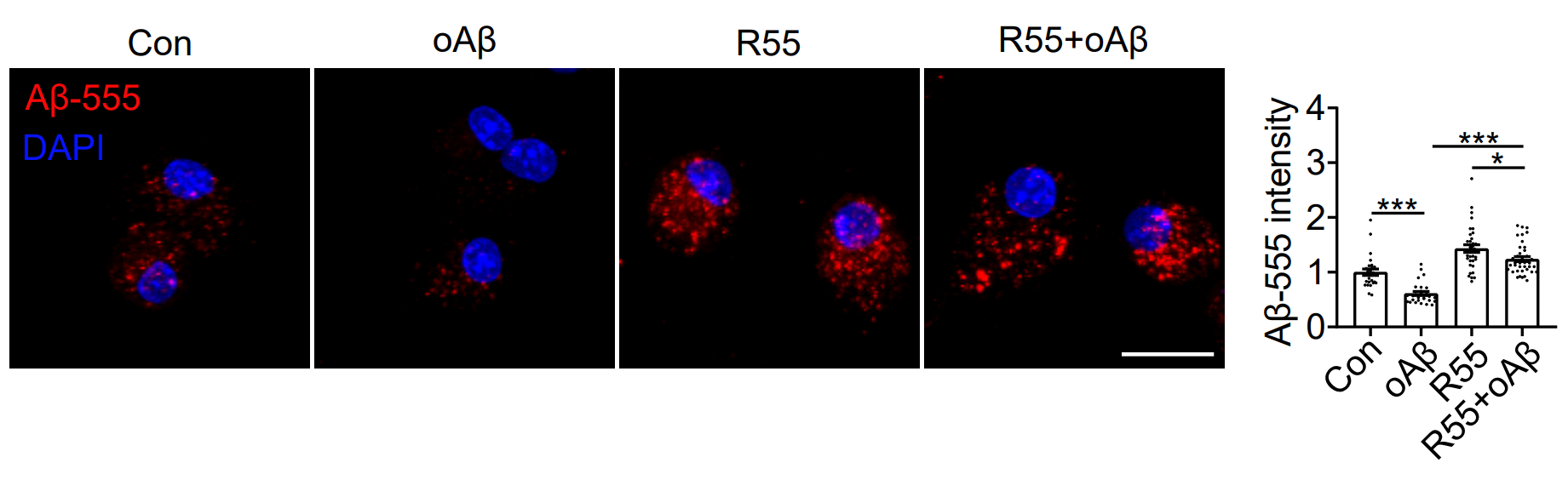


**Supp Fig. 4 R55 enhances the Aβ uptake by Aβ-exposed microglia.** Primary microglia were co-treated with oAβ and R55 for 12 h. Then cells were incubated with Aβ-555 for 30 min and counterstained by DAPI. Scale bar = 20 μm. n > 100, * *p* < 0.05 and *** *p* < 0.001 by two-way ANOVA.


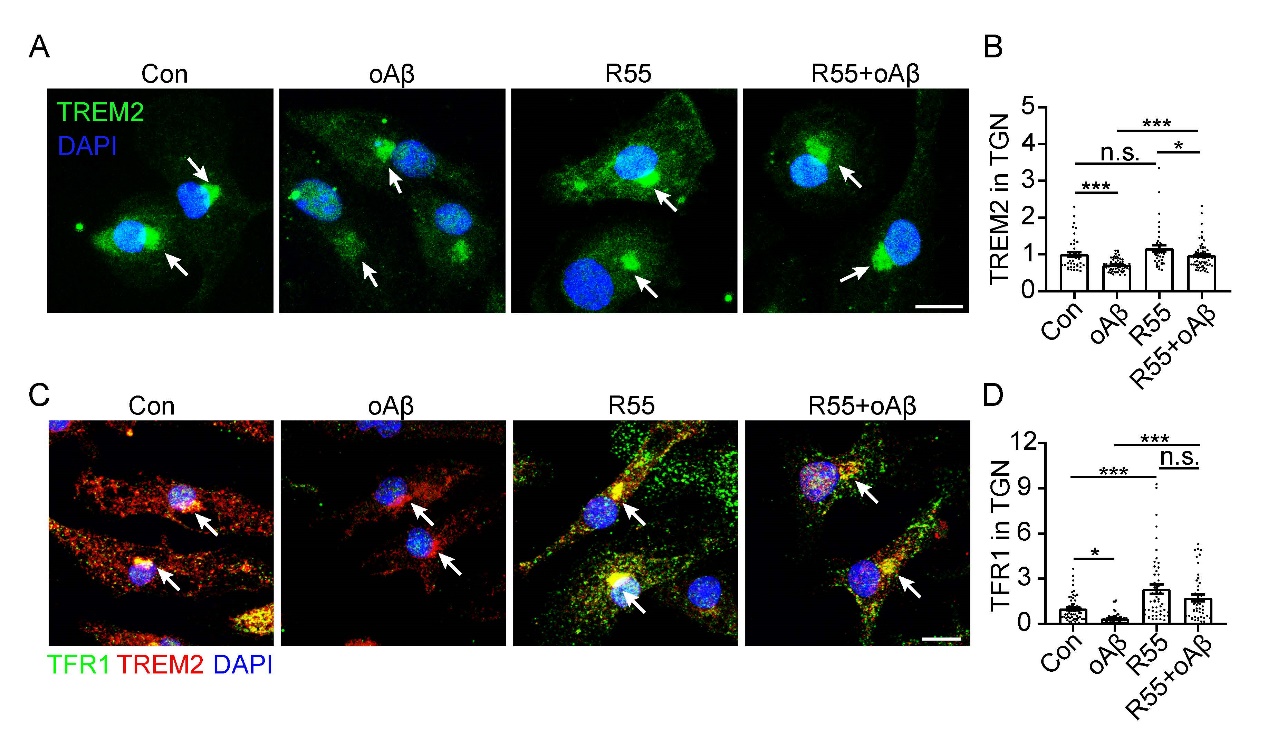


**Supp Fig. 5 R55 reverses the abnormal distribution of TREM2** **or TFR1 in Aβ-exposed microglia.** Primary microglia were co-treated with oAβ and R55 for 12 h. (**A** and **C**) Cells were probed with anti-TREM2 (**A**) or TFR1 (**C**) antibodies and counterstained by DAPI. The arrows indicate the location of the trans Golgi network (TGN). Scale bar = 10 μm. TREM2 (**B**) or TFR1(**D**) intensity in the location of trans Golgi network in Aβ-exposed microglia in panel **A** and **B**. n > 100, * *p* < 0.05 and *** *p* < 0.001 by two-way ANOVA. n.s. indicated no significant difference.


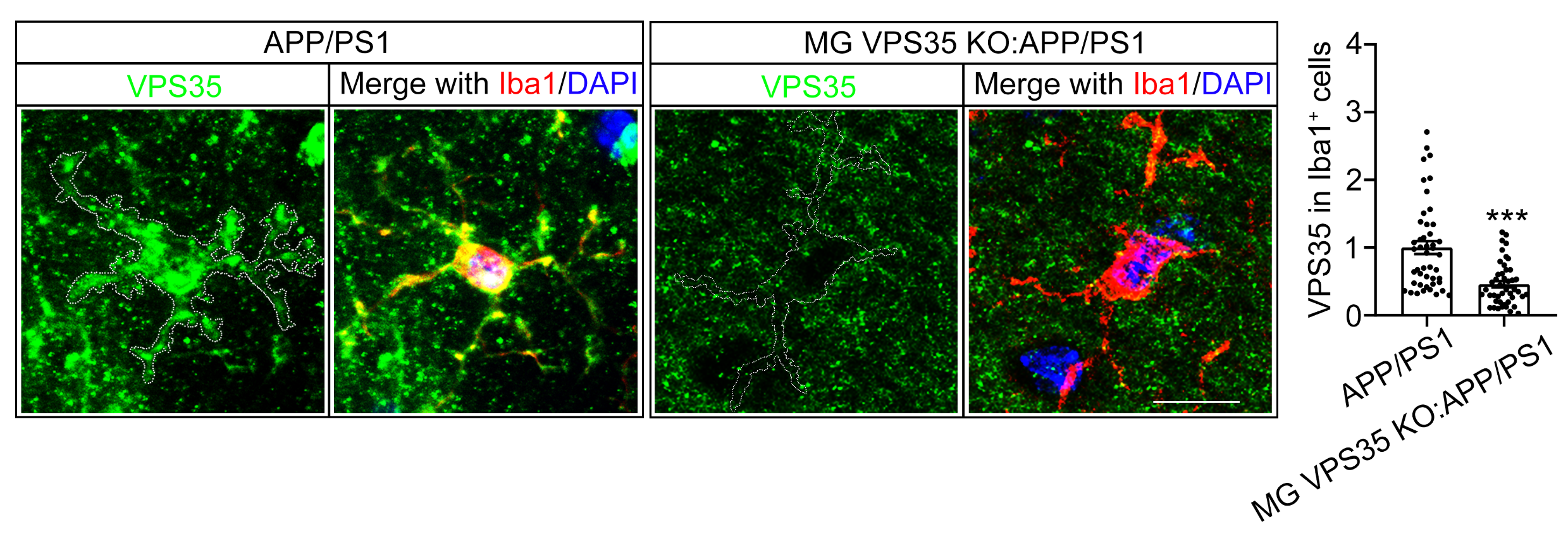


**Supp Fig. 6 Validation of the VPS35 knockout in microglia of APP/PS1 mice.** After tamoxifen injection, brain sections from MG VPS35 KO:APP/PS1 mice in CA1 region were labeled with anti-Iba1 and anti-VPS35 antibodies. Scale bar = 10 μm. n > 50, *** *p* < 0.001 by Student's t-test.


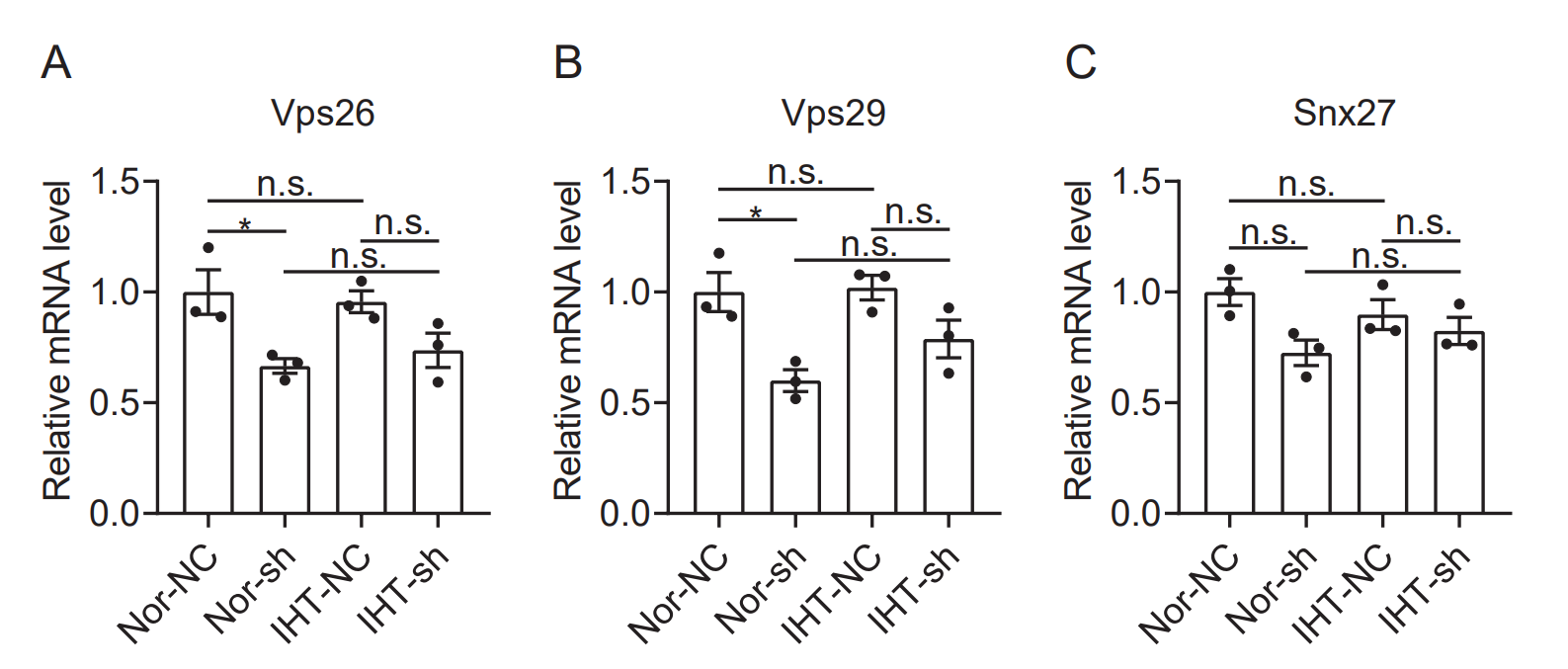


**Supp Fig. 7 IHT has no significant effect on *Vps26*, *Vps29* and *Snx27* in Aβ-exposed sh*Tfeb* BV2.** sh*Tfeb* BV2 cells were exposed to oAβ and then treated with IHT. The mRNA level of *Vps26*, *Vps29* and *Snx27* were measured using qRT-PCR. n = 3, * *p* < 0.05 by two-way ANOVA. n.s. indicated no significant difference. Nor, normoxia.


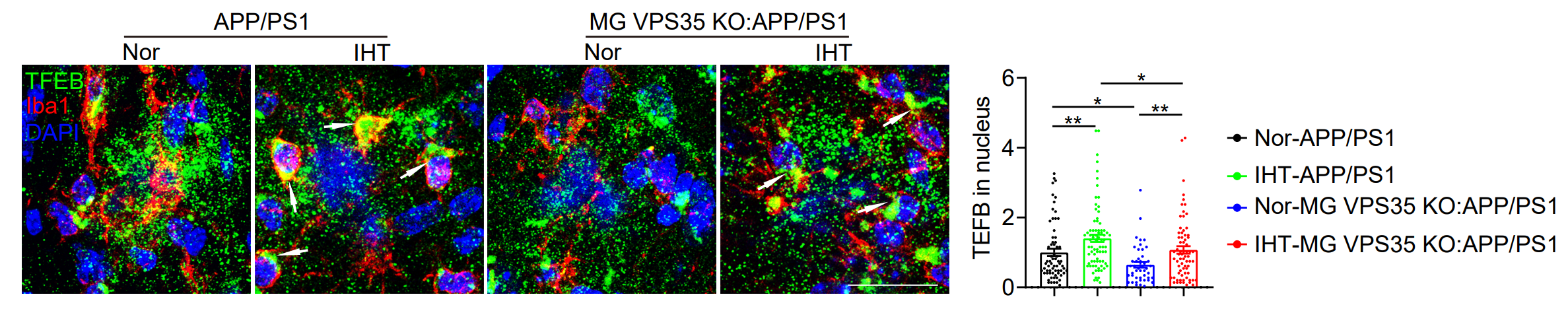


**Supp Fig. 8 IHT upregulates TFEB in PAM of MG VPS35 KO:APP/PS1 mice.** Brain sections of IHT-treated mice in CA1 region were labeled with anti-TEFB and anti-Iba1 antibodies, followed with counterstaining by DAPI. Scale bar = 20 μm. n > 50, * *p* < 0.05 and ** *p* < 0.01 by two-way ANOVA. Nor, normoxia.
