# Supplementary material for: Intermittent hypoxia training enhances Aβ endocytosis by plaque associated microglia via VPS35-dependent TREM2 recycling in murine Alzheimer’s disease
Source: Alzheimers Res Ther. 2024 Jun 3;16:121. doi: 10.1186/s13195-024-01489-6 (PMC11145795; doi:10.1186/s13195-024-01489-6)
Supplement: Supplementary file 2 — Supplementary Material 2 [file 13195_2024_1489_MOESM2_ESM.docx]

WB Bands

Figure 3A VPS35 90 kDa


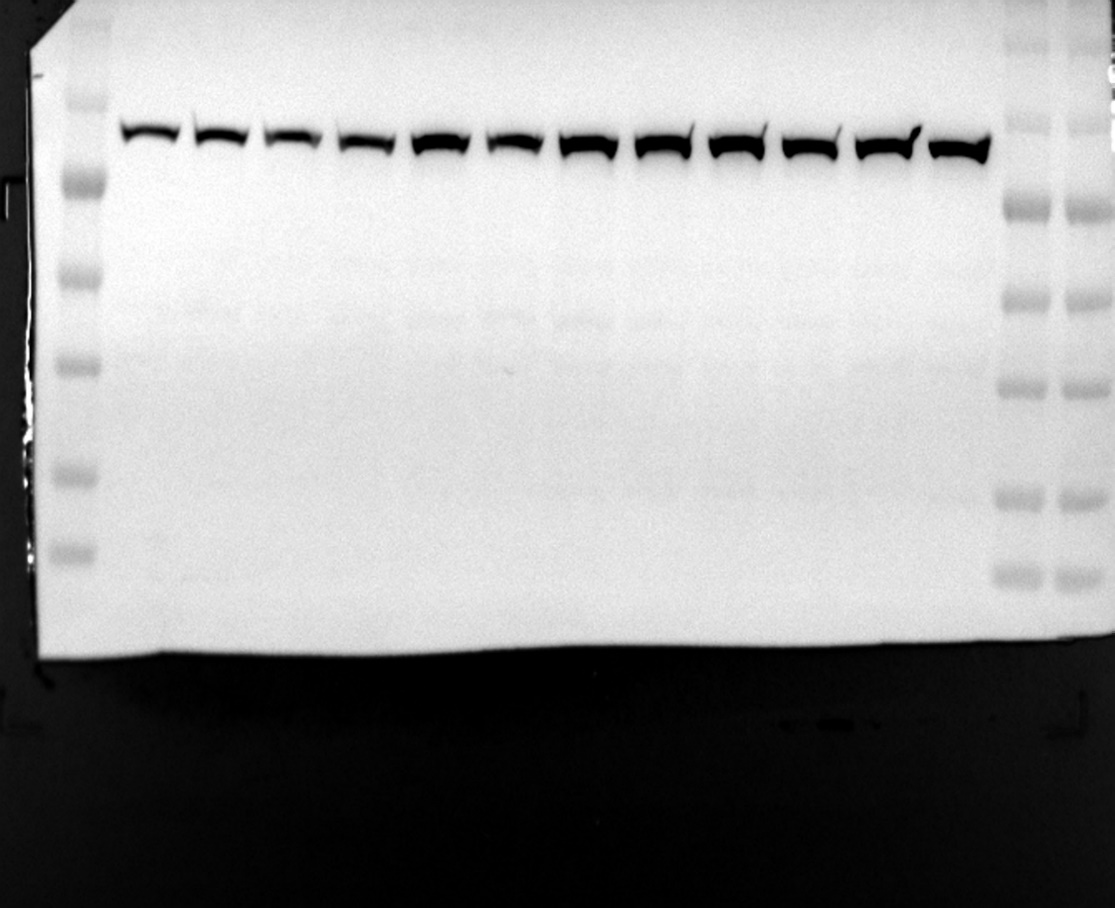


VPS35 90 kDa

150 kDa

100 kDa

70 kDa

55 kDa

40 kDa

25 kDa

150 kDa

100 kDa

70 kDa

55 kDa

40 kDa

25 kDa

15 kDa

15 kDa

Nor-TG

IHT-TG

Figure 3A β-actin 42 kDa


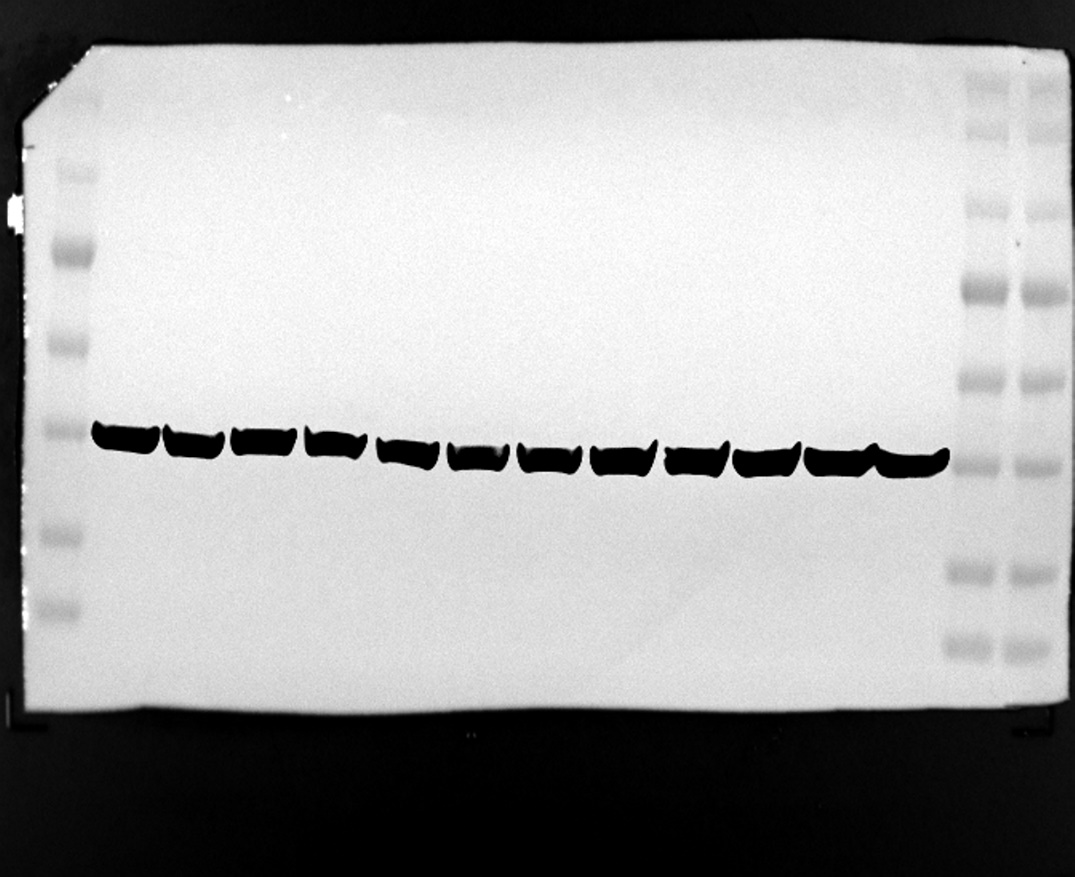


β-actin 42 kDa

150 kDa

100 kDa

70 kDa

55 kDa

40 kDa

25 kDa

150 kDa

100 kDa

70 kDa

55 kDa

40 kDa

25 kDa

15 kDa

15 kDa

Nor-TG

IHT-TG

180 kDa

Nor-TG

IHT-TG

Figure 3H VPS35 90 kDa


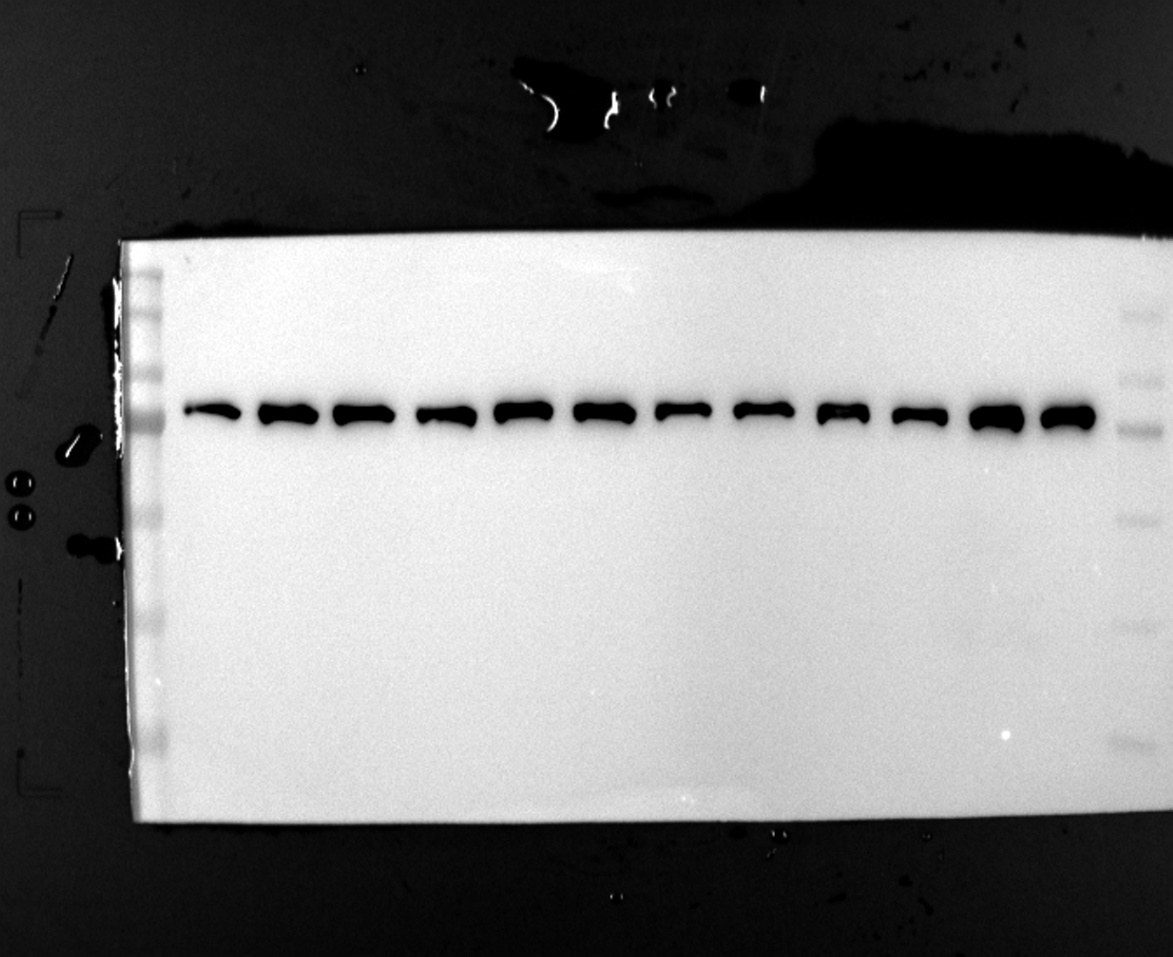


150 kDa

180 kDa

100 kDa

150 kDa

100 kDa

70 kDa

70 kDa

55 kDa

55 kDa

40 kDa

40 kDa

25 kDa

25 kDa

VPS35 90 kDa

Nor oAβ IHT IHT+oAβ

Figure 3H β-actin 42 kDa


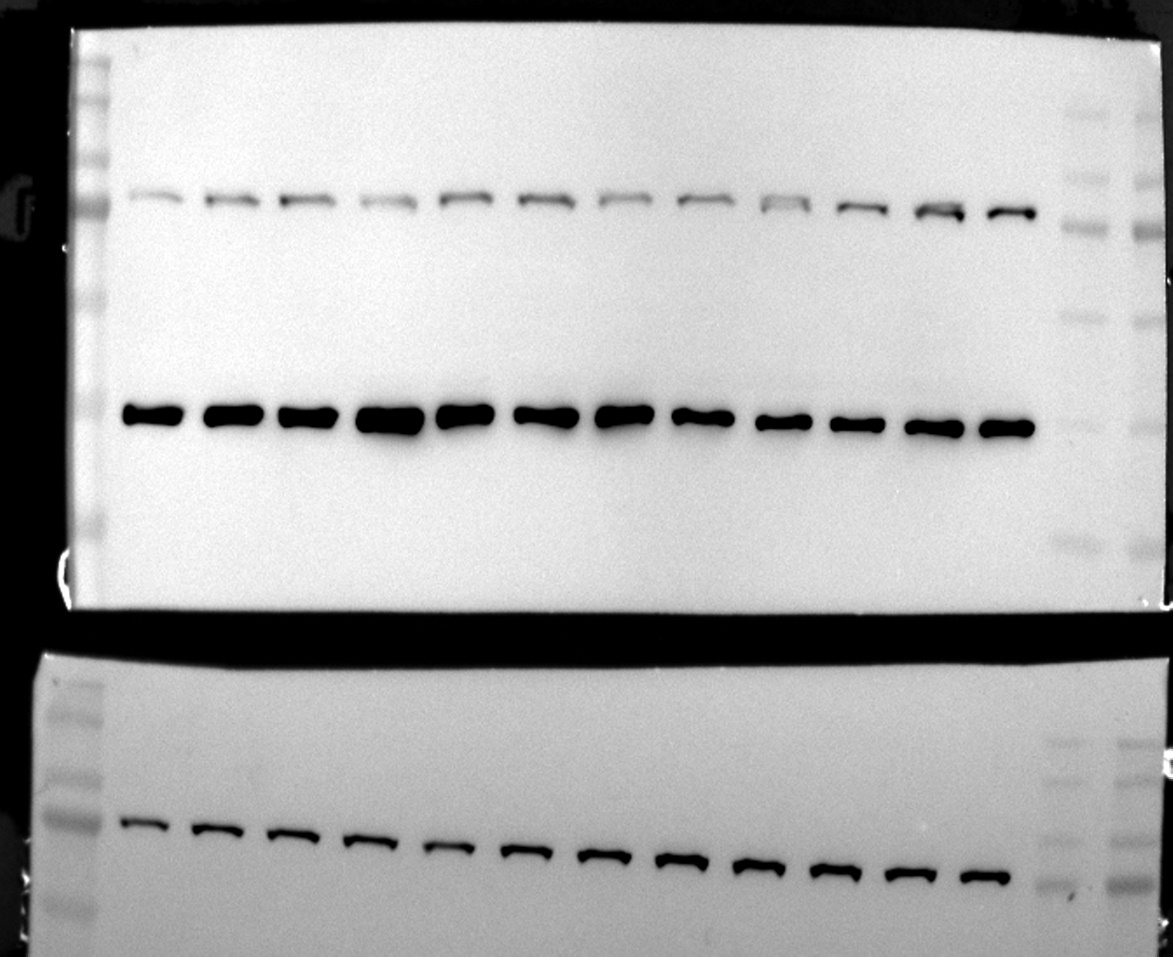


β-actin 42 kDa

Nor oAβ IHT IHT+oAβ

150 kDa

100 kDa

70 kDa

55 kDa

40 kDa

25 kDa

180 kDa

150 kDa

100 kDa

70 kDa

55 kDa

40 kDa

25 kDa

Figure 8E TFEB 65kDa


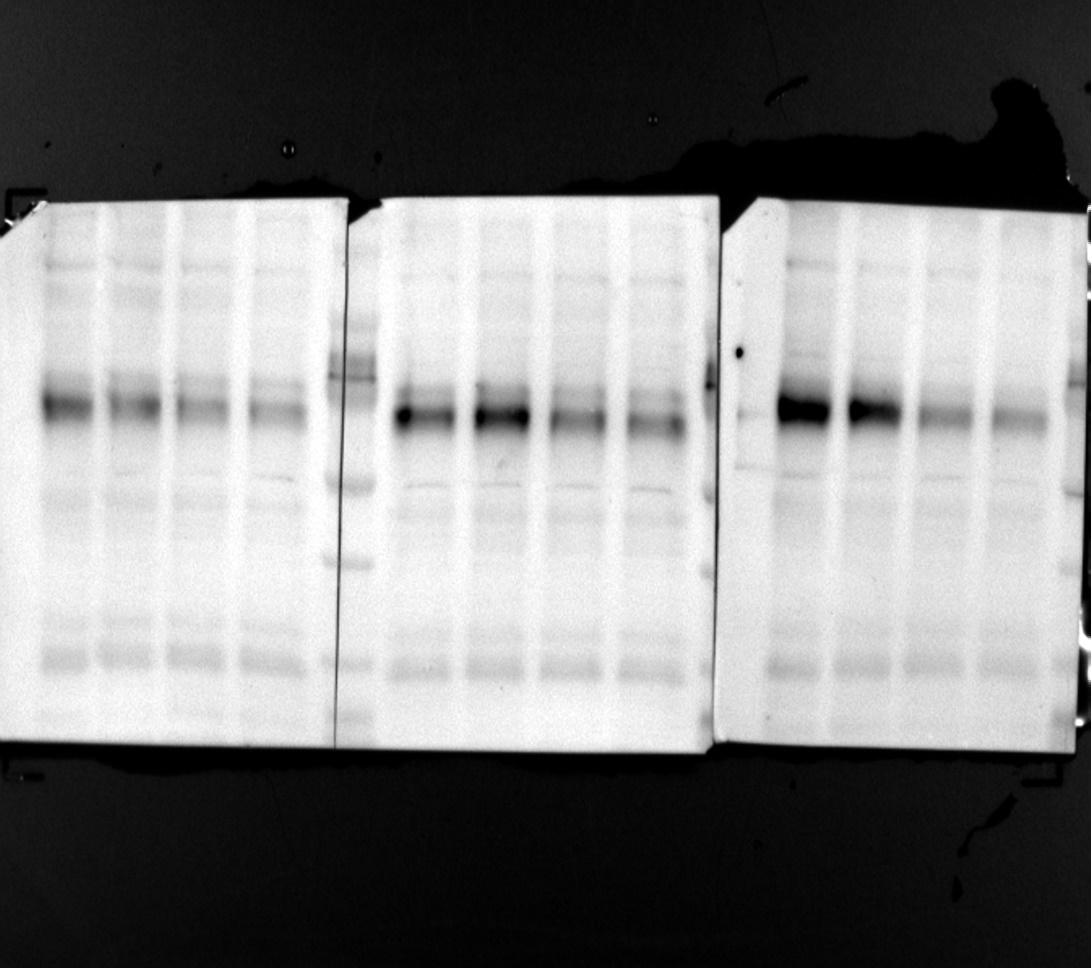


TFEB 65 kDa

Nor IHT sh IHT-sh

100 kDa

70 kDa

55 kDa

40 kDa

25 kDa

130 kDa

180 kDa

100 kDa

70 kDa

55 kDa

40 kDa

25 kDa

130 kDa

180 kDa

Figure 8E VPS35 90kDa


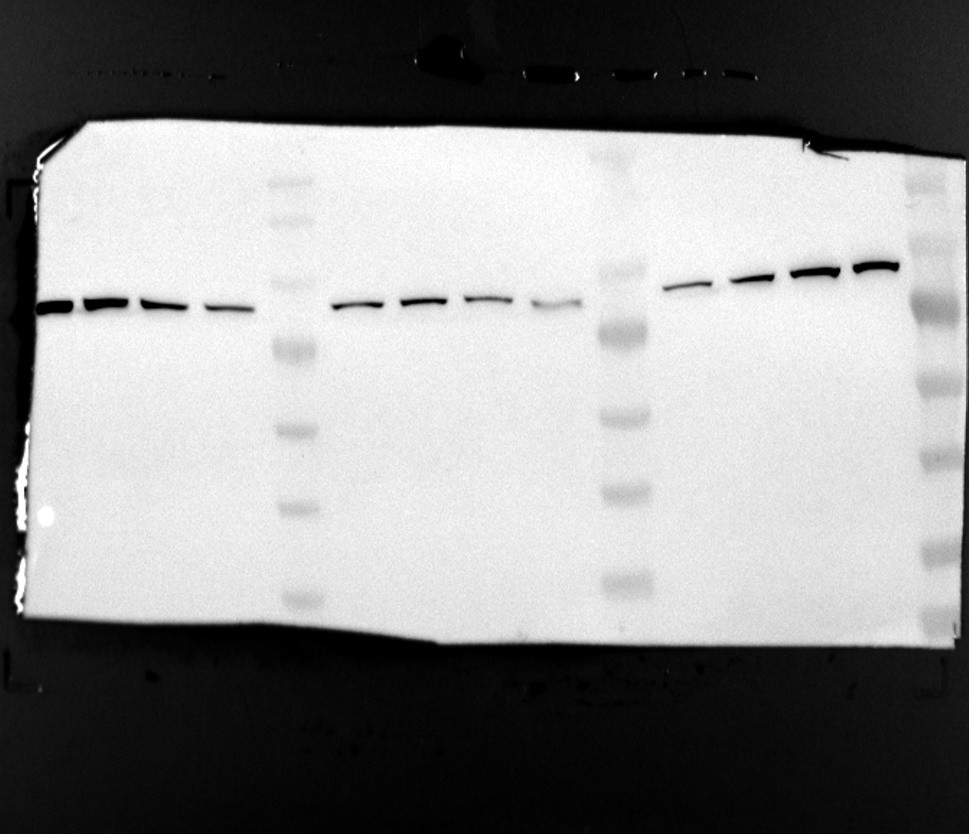


100 kDa

70 kDa

55 kDa

40 kDa

VPS35 90 kDa

Nor IHT sh IHT-sh

25 kDa

130 kDa

180 kDa

100 kDa

70 kDa

55 kDa

40 kDa

25 kDa

130 kDa

180 kDa

Figure 8E β-actin 42kDa


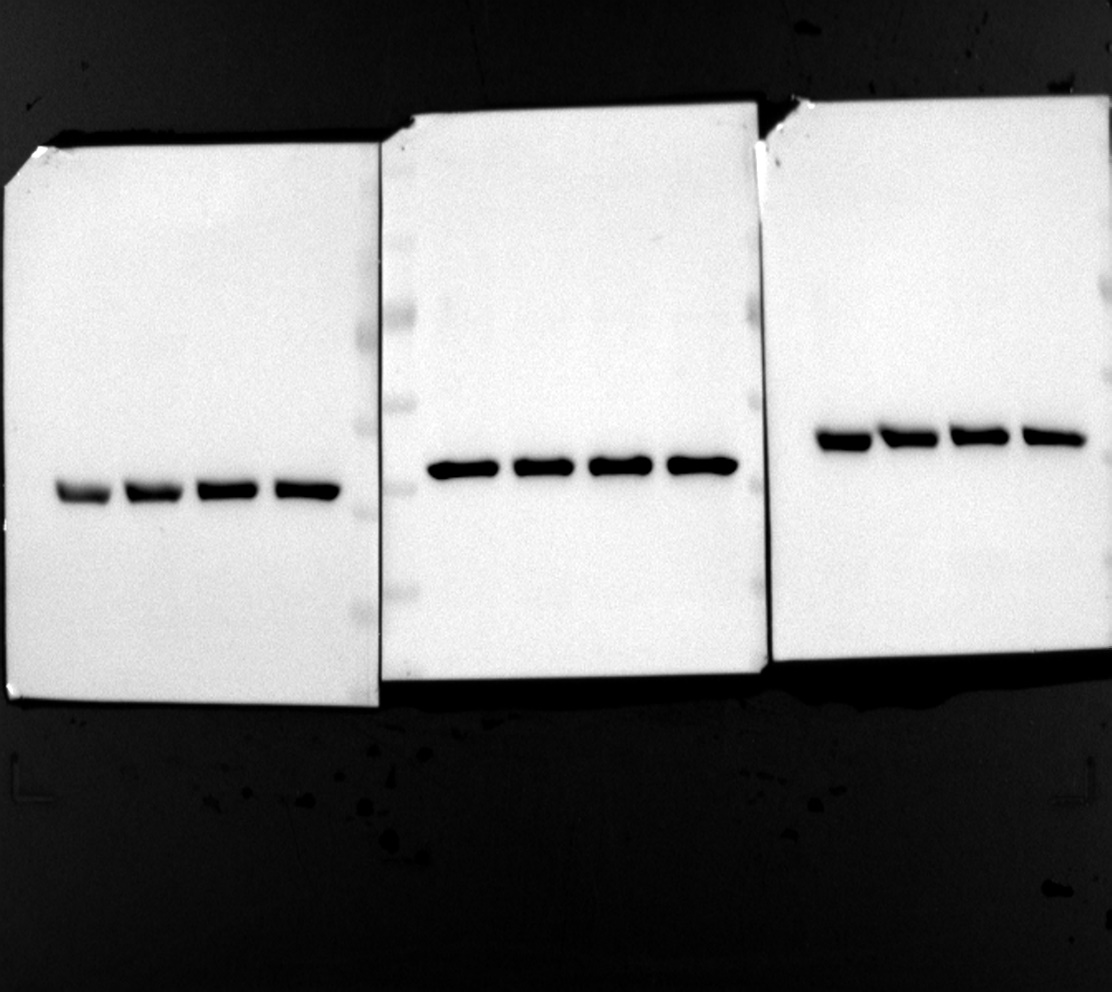


100 kDa

70 kDa

55 kDa

40 kDa

25 kDa

130 kDa

180 kDa

100 kDa

70 kDa

55 kDa

40 kDa

25 kDa

130 kDa

180 kDa

β-actin 42 kDa

Nor IHT sh IHT-sh

Agarose gel electropherogram

Figure 7B


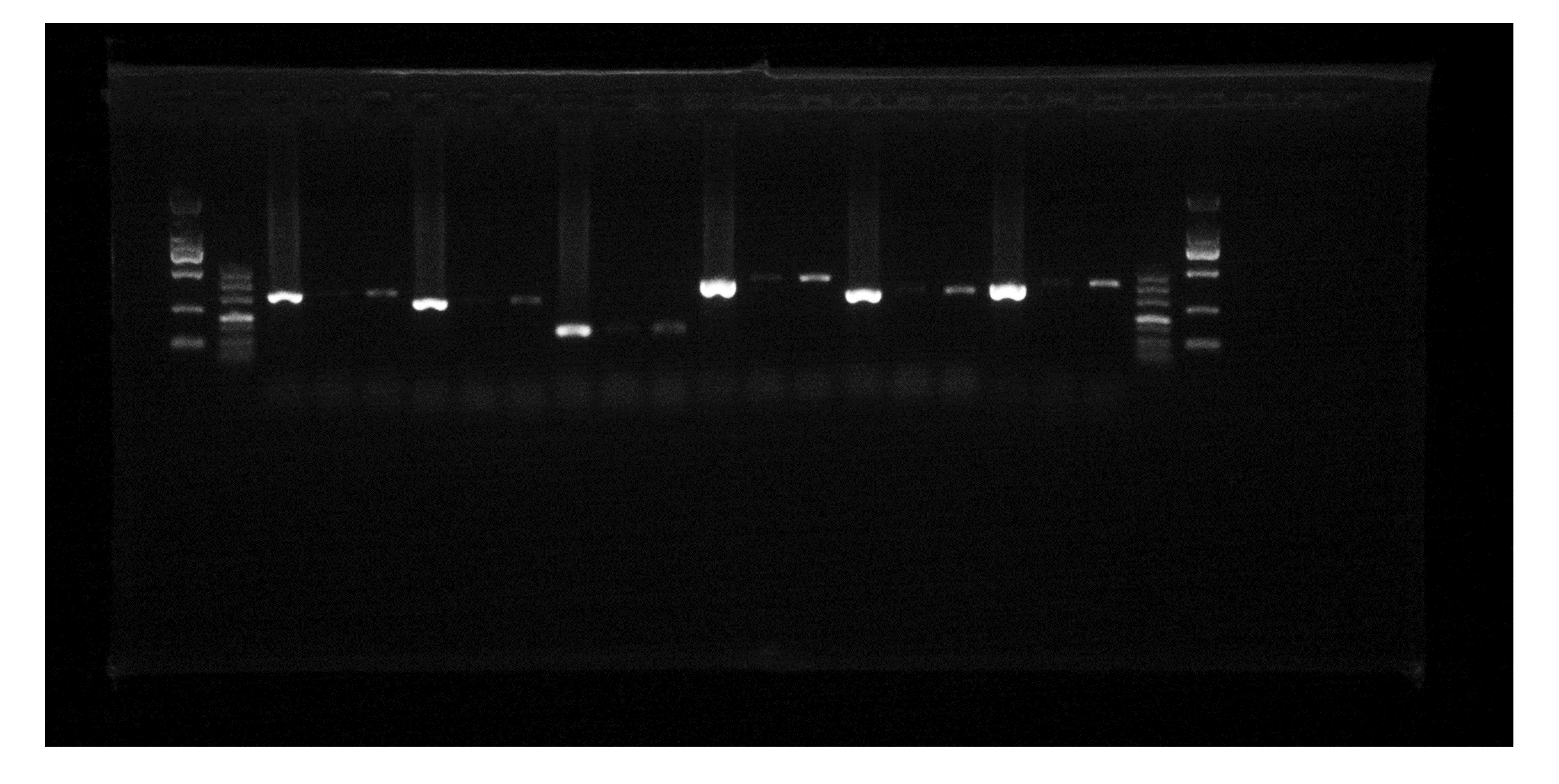


Marker

Marker

Marker

Marker

500 bp

200 bp

100 bp

500 bp

200 bp

100 bp

Input

IgG

TFEB

Input

IgG

TFEB

Input

IgG

TFEB

Input

IgG

TFEB

Input

IgG

TFEB

Input

IgG

TFEB

①

②

③

④

⑤

⑥

① mVps35(-146/165) 311 bp

② mVps35(-155/118) 273 bp

③ mVps35(148/278) 131 bp

④ mVps35(96/504) 409 bp

⑤ mVps35(341/660) 320 bp

⑥ mVps35(347/713) 367bp

TFEB potential binding site (-3/7)

TFEB potential binding site (204/213)

TFEB potential binding site (628/637)
